# Ultrasound-targeted microbubble cavitation enhances anti–PD-L1 therapy in TNBC via eNOS-mediated reoxygenation

**DOI:** 10.1172/jci.insight.198349

**Published:** 2026-04-07

**Authors:** Zhiyu Zhao, Li Ba, Siwei Li, Jianxin Wang, Yuzhou Luo, Sihan Wang, Yan Jin, Changjun Wu

**Affiliations:** 1 Department of Ultrasound, First Affiliated Hospital of Harbin Medical University, Harbin, China.; 2 Laboratory of Medical Genetics, Harbin Medical University, Harbin, China.; 3 Key Laboratory of Preservation of Human Genetic Resources and Disease Control in China, Ministry of Education, Harbin Medical University, Harbin, China.; 4 Department of Breast Surgery, Harbin Medical University Cancer Hospital, Harbin, China.; 5 Department of Neurology, Brain Ultrasound, First Affiliated Hospital of Harbin Medical University, Harbin, China.; 6 State Key Laboratory of Zone Cardiovascular Diseases in China, Harbin Medical University, Harbin, China.

**Keywords:** Immunology, Oncology, Vascular biology, Breast cancer, Cancer immunotherapy, Endothelial cells

## Abstract

Hypoxia critically restricts the effectiveness of immunotherapy in triple-negative breast cancer (TNBC). Comprehensive bioinformatics analyses have demonstrated that highly hypoxic TNBC tumors exhibited elevated T cell exhaustion, increased immune checkpoint molecule expression, and diminished responsiveness to immune checkpoint blockade (ICB). Consequently, strategies aimed at alleviating tumor hypoxia may effectively augment ICB therapy. Although ultrasound-targeted microbubble cavitation (UTMC) has been shown to reduce tumor hypoxia, the precise molecular mechanisms remain unclear. Here, we provide evidence that UTMC activated endothelial nitric oxide synthase (eNOS) through G protein–coupled signaling, resembling pathways induced by fluid shear stress. UTMC-induced eNOS activation was largely Ca^2+^ dependent and resulted in increased nitric oxide production. Enhanced nitric oxide generation was associated with improved tumor perfusion and reduced hypoxia. Combining UTMC with anti–PD-L1 therapy markedly improved the tumor immune microenvironment, characterized by increased CD8^+^ T cell infiltration, reduced T cell exhaustion, diminished regulatory T cell infiltration, increased macrophage polarization from an M2 to M1 phenotype, and elevated production of proinflammatory cytokines. Collectively, our findings identified UTMC as a promising adjunctive therapeutic approach to mitigate hypoxia and enhance the efficacy of anti–PD-L1 immunotherapy in TNBC. These results support further translational evaluation of UTMC-based combination strategies in hypoxic TNBC.

## Abstract 2

Ultrasound-targeted microbubble cavitation activates eNOS via G protein signaling, alleviating hypoxia and enhancing immunotherapy efficacy in triple-negative breast cancer.

## Introduction

Triple-negative breast cancer (TNBC) is characterized by the absence of estrogen receptor, progesterone receptor, and human epidermal growth factor receptor-2 expression, and is associated with aggressive clinical progression, limited therapeutic options, and poor prognosis. Current standard treatments primarily involve radiotherapy, chemotherapy, and emerging immunotherapy, but clinical outcomes remain suboptimal due to rapid disease progression and frequent development of therapeutic resistance (1). Hypoxia, a hallmark feature of the TNBC tumor microenvironment (TME), markedly exacerbates disease aggressiveness by promoting cancer cell survival, invasion, and metastasis, and critically contributes to resistance to multiple therapies, including immunotherapy (2–4). Under hypoxic conditions, substantial changes occur within the tumor immune microenvironment, including increased infiltration of immunosuppressive cells such as regulatory T cells (Tregs) and M2-type tumor-associated macrophages (TAMs), impaired cytotoxic T cell function, induction of T cell exhaustion, and elevated expression of inhibitory immune checkpoint molecules, collectively facilitating immune escape and immunotherapeutic resistance (5, 6). Consequently, strategies aimed at alleviating tumor hypoxia represent a rational adjunctive strategy to enhance immunotherapy efficacy in TNBC.

Ultrasound-targeted microbubble cavitation (UTMC) utilizes low-intensity ultrasound to induce microbubble cavitation within tissues, generating localized mechanical effects such as radiation forces, microstreaming, shock waves, and micro-jets (7). Depending on the acoustic parameters, UTMC encompasses a continuum of cavitation behaviors ranging from stable oscillation to transient inertial events. These biomechanical effects have been reported to enhance vascular permeability, improve blood perfusion, alleviate ischemia, and modulate local vascular function, thereby contributing to diverse therapeutic applications (8–10). Recently, UTMC-induced enhancement of tissue reperfusion and partial alleviation of hypoxia has been termed “sonoreperfusion” (11). This process is proposed to involve shear stress–mediated ATP release, which subsequently promotes the endogenous production of vasodilators, particularly nitric oxide (NO), resulting in local microvascular dilation and enhanced blood perfusion (12).

Although sonoreperfusion has shown promise in preclinical studies and is under clinical evaluation (13–16), the exact molecular pathways underlying this phenomenon remain incompletely defined. Traditionally, UTMC-induced vasodilation and improved perfusion have been interpreted primarily through the fluid shear stress model, which involves 2 principal G protein subunit–related signaling pathways: the GNAS/PKA-mediated and the GNAQ/GNA11/PDK1/AKT-mediated pathways. Both pathways synergistically activate endothelial NO synthase (eNOS), thereby increasing NO production, leading to vasodilation and enhanced local blood perfusion (17, 18). However, notable differences in mechanical stimuli parameters, such as shear angle, intensity, and dynamics, between UTMC-induced forces and classical fluid shear stress raise substantial uncertainties regarding the congruence of their signaling pathways (7). Therefore, direct experimental validation of UTMC-triggered eNOS activation and its upstream signaling mechanisms is required.

In this study, we systematically characterized the immunological features of TNBC based on the expression profiles of hypoxia-related genes (HRGs), using data from The Cancer Genome Atlas (TCGA) and Gene Expression Omnibus (GEO). Through comprehensive analyses, we examined functional signatures via immune-related profiles using the R package IOBR, immune checkpoint gene (ICG) expression, T cell functional states, and performed Tumor Immune Dysfunction and Exclusion (TIDE) analysis. Our findings provided evidence that alleviation of tumor hypoxia may effectively enhance the therapeutic benefit of immune checkpoint blockade (ICB) in TNBC. Furthermore, we explored the molecular mechanisms by which UTMC activates eNOS, leading to vasodilation and improved tumor perfusion. To our knowledge, this study provides the first direct evidence linking UTMC-induced eNOS activation to specific G protein–mediated signaling pathways that closely resemble those triggered by fluid shear stress (18, 19). Finally, in murine TNBC models, we demonstrated that combining UTMC-induced hypoxia alleviation with anti–PD-L1 therapy substantially improved therapeutic responses, supporting a translational strategy to overcome hypoxia-driven immunotherapy resistance in TNBC.

## Results

### Immunological characterization of TNBC stratified by hypoxia status in TCGA and GEO cohorts.

Immune-related functional signatures differed significantly between high- and low-hypoxia TNBC tumors. As shown in Supplemental Figure 1A (supplemental material available online with this article; https://doi.org/10.1172/jci.insight.198349DS1), signatures associated with ICB resistance, cancer-associated fibroblasts (CAFs), EMT, myeloid-derived suppressor cells (MDSCs), pan-fibroblast TGF-β response (Pan-F-TBRs), TAMs, TGF-β (TGFB1) family members and receptors, T cell exhaustion, and Treg activity were significantly elevated in the high-hypoxia group compared with the low-hypoxia group in both TCGA and GEO TNBC cohorts.

**Figure 1:**
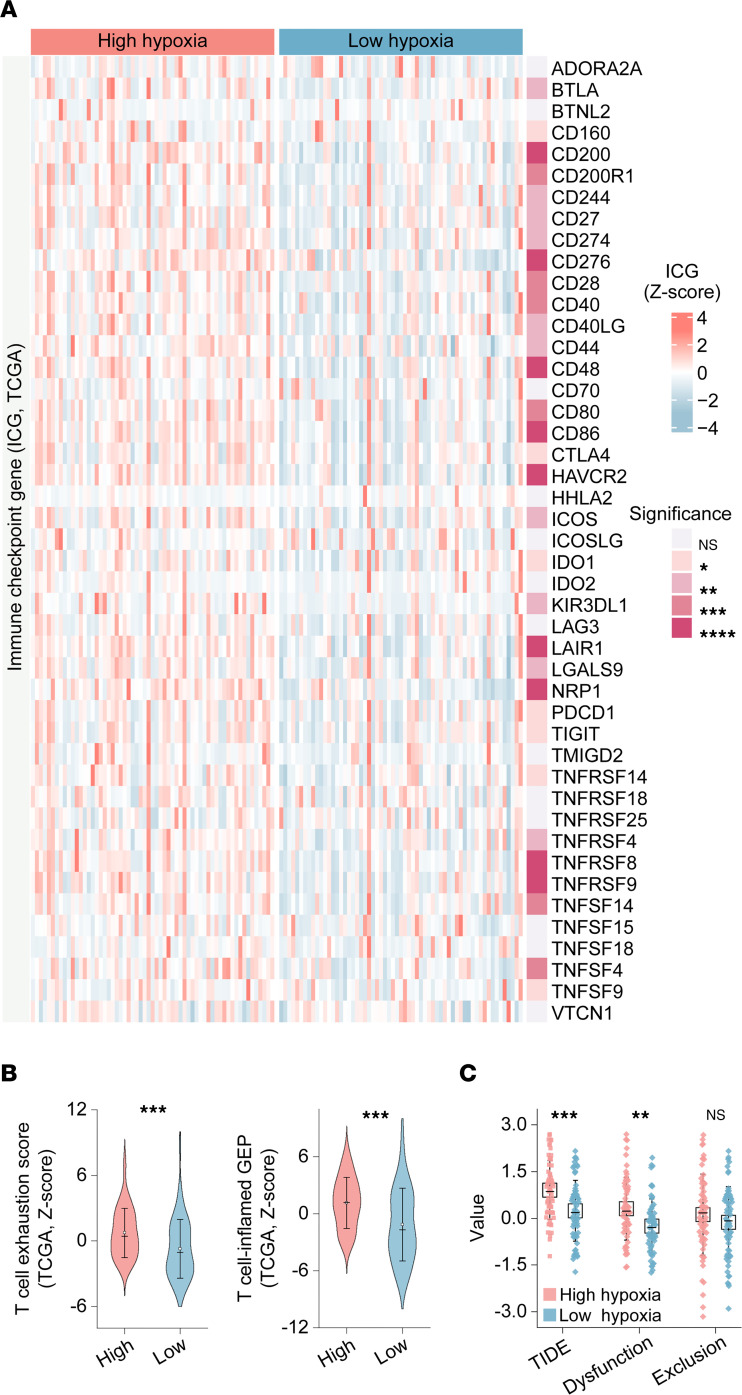
Immunological characterization of patients with TNBC stratified by hypoxia status. (**A**) Heatmap depicting normalized expression of 45 ICGs in TNBC samples stratified by high- and low-hypoxia status from the TCGA cohort. (**B**) Comparison of T cell exhaustion scores and T cell–inflamed GEPs between high- and low-hypoxia TNBC groups in TCGA cohort. (**C**) TIDE analysis comparing TIDE score, T cell dysfunction, and T cell exclusion between high- and low-hypoxia groups in TNBC samples from TCGA cohort. Statistical analyses were performed using Wilcoxon’s rank-sum test. NS, no significance. *P < 0.05, **P < 0.01, ***P < 0.001, ****P < 0.0001.

Consistent with these findings, analysis of ICGs revealed significant upregulation in high-hypoxia tumors (Figure 1A and Supplemental Figure 1B). Specifically, key ICGs known to mediate immunosuppression and T cell exhaustion, including CD274 (PD-L1), CD276, CTLA4, HAVCR2 (Tim-3), LAG3, TNFRSF4, TNFRSF8, TNFRSF9, LGALS9, LAIR1, NRP1, CD27, CD40, CD44, CD80, and CD86, exhibited significantly higher expression levels in the high-hypoxia group across both TCGA- and GEO-TNBC cohorts. PDCD1 (PD-1) and CD70 exhibited significant differential expression only in the TCGA cohort. Collectively, these findings indicate that high-hypoxia TNBC tumors are associated with an immunosuppressive microenvironment characterized by elevated immune checkpoint expression, enhanced fibroblast-related signatures, increased EMT activity, and enrichment of immunosuppressive cell populations.

### T cell functional characteristics of TNBC stratified by hypoxia status in TCGA and GEO cohorts.

As shown in Figure 1B and Supplemental Figure 1, C–E, both *Z*-score normalization and single-sample gene set enrichment analysis (ssGSEA) demonstrated that high-hypoxia TNBC from both TCGA and GEO cohorts exhibited significantly elevated T cell exhaustion scores and increased T cell–inflamed gene expression profiles (GEPs) compared with low-hypoxia tumors. These findings indicate an exhausted T cell phenotype in high-hypoxia tumors.

### Clinical relevance of hypoxia stratification in TNBC from the TCGA-TNBC cohort.

As shown in Figure 1C, TIDE analysis revealed significant differences between high- and low-hypoxia groups in the TCGA-TNBC cohort. High-hypoxia tumors exhibited significantly higher TIDE scores, consistent with reduced predicted responsiveness to ICB. Additionally, T cell dysfunction scores were significantly elevated in the high-hypoxia group compared with the low-hypoxia group. No significant differences were observed in T cell exclusion scores between the 2 groups. Collectively, these findings support an association between tumor hypoxia and immune dysfunction, accompanied by higher resistance to ICB. Cox proportional hazards analysis further demonstrated that hypoxia status was not significantly associated with overall survival (OS; HR = 0.969, *P* = 0.947), disease-specific survival (DSS; HR = 1.06, *P* = 0.916), or progression-free interval (PFI) (HR = 0.619, *P* = 0.264) in the TCGA cohort (Supplemental Table 1). Thus, although hypoxia status was not an independent prognostic factor, it was linked to immune dysfunction and predicted immunotherapy resistance in TNBC.

### Ultrasound-mediated microbubble disruption in vitro.

**Figure 2:**
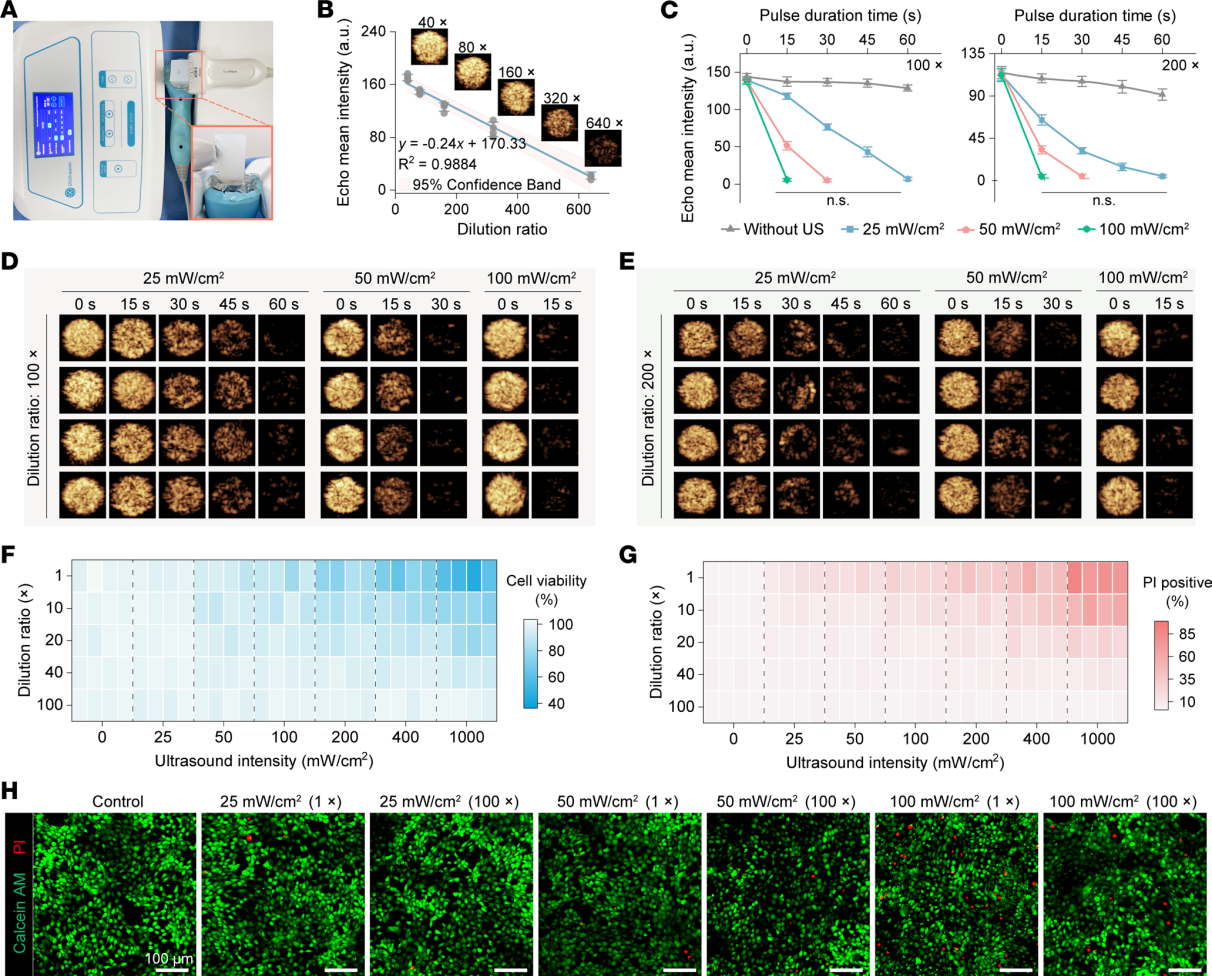
Evaluation of UTMC-induced microbubble disruption and endothelial cell damage. (**A**) Experimental setup illustrating the arrangement of equipment used for UTMC-induced microbubble rupture. (**B**) Linear regression plot depicting the relationship between mean acoustic intensity and the dilution ratio of microbubble contrast agent (representative images from 4 independent experiments are shown; *n* = 4 per group). (**C**–**E**) CEUS images and corresponding temporal curves of mean acoustic intensity at specific time points before and after ultrasonic irradiation of microbubble contrast agent (*n* = 4 per group). (**F** and **G**) Heatmaps showing the cell viability and propidium iodide–positive (PI-positive) rates of HUVECs following UTMC treatment at the indicated ultrasound intensities and microbubble dilution ratio (*n* = 4 per group). (**H**) Representative Calcein AM/PI staining images of HUVECs treated with UTMC at the indicated ultrasound intensities and microbubble dilution ratio. Green fluorescence indicates viable cells, whereas red fluorescence indicates membrane-compromised cells. Representative images from 3 independent experiments are shown. Scale bars: 100 μm. Statistical analyses were performed using 1-way ANOVA with Tukey’s post hoc test. NS, no significance.

To quantitatively assess UTMC-induced microbubble disruption, echo mean intensity (EMI) within the microbubble region was measured using contrast-enhanced ultrasound (CEUS). EMI served as a surrogate for microbubble concentration. As illustrated in Figure 2A, microbubbles with varying dilution ratios were placed into wells of a custom mold. Following region of interest–based (ROI-based) quantitative analysis using QLAB software, a linear regression was performed to correlate microbubble concentration with EMI (Figure 2B). The linear regression analysis demonstrated a strong correlation (*R*^2^ = 0.9884), supporting the use of EMI as a quantitative measure of microbubble concentration.

Next, to evaluate the effects of different UTMC exposure conditions, microbubble disruption under varying ultrasound intensities at constant microbubble concentrations. As shown in Figure 2, C–E, microbubbles diluted 100-fold (100×) and 200× were exposed to ultrasound at an intensity of 25 mW/cm^2^ for 60 seconds, 50 mW/cm^2^ for 30 seconds, or 100 mW/cm^2^ for 15 seconds. Each condition resulted in complete microbubble disruption, with EMI values approaching zero and no significant differences observed among groups. These findings demonstrate that equivalent levels of microbubble disruption can be achieved under different combinations of ultrasound intensity and exposure duration when initial microbubble concentration is controlled.

### UTMC-induced cellular injury in human umbilical vein endothelial cells.

As shown in Figure 2F, the viability of human umbilical vein endothelial cells (HUVECs) decreased notably following UTMC treatment, with greater reductions observed at higher ultrasound intensities and microbubble concentrations. Consistent with these changes, the proportion of propidium iodide–positive (PI-positive) cells increased correspondingly (Figure 2G). These data demonstrate a dose-dependent increase in membrane injury with escalating ultrasound intensity and microbubble concentration. Notably, at a microbubble dilution of 100×, exposure to ultrasound intensities up to 1000 mW/cm^2^ resulted in minimal cell injury, as indicated by cell viability greater than 95% and a PI-positive rate below 5%. These results justified the use of the 100× microbubble dilution in subsequent mechanistic experiments. Consistent with the quantitative analyses, Calcein AM/PI staining (Figure 2H) showed predominantly viable (green) cells with minimal PI-positive staining under the 100× dilution condition across ultrasound intensities, whereas increased PI-positive cells were observed under higher ultrasound intensity and microbubble concentration.

### UTMC promotes endothelial mediator release and eNOS phosphorylation in HUVECs.

**Figure 3:**
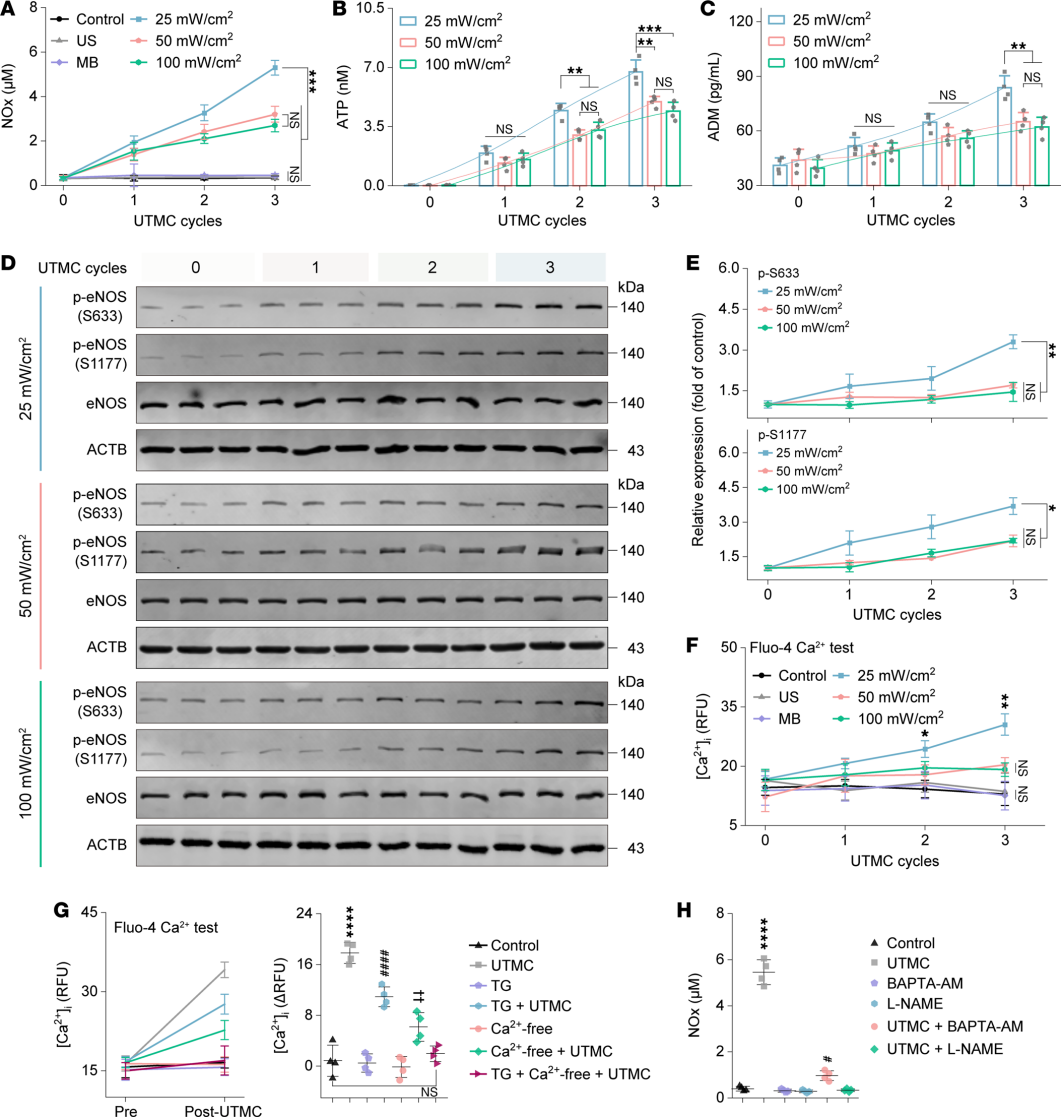
eNOS activation induced by mechanical effects of UTMC. (**A**) Concentration of NOx in culture supernatants after UTMC treatment at different cycles (*n* = 4 per group). (**B** and **C**) Concentration of ATP and ADM in culture supernatants following UTMC treatment with different exposure cycles (*n* = 4 per group). (**D** and **E**) Western blot bands and corresponding quantitative analysis illustrating the phosphorylation status of eNOS in HUVECs following UTMC treatment with different exposure cycles (*n* = 3 per group). The same samples were run in a separate gel for detecting phosphorylated eNOS. (**F**) Fluo-4–based quantification of cytosolic Ca^2+^ following UTMC treatment with different exposure cycles. RFU of Fluo-4 were recorded after each UTMC cycle (*n* = 4 per group). (**G**) Fluo-4–based quantification of cytosolic Ca^2+^ dynamics following 3 UTMC cycles. Left: Relative RFU of Fluo-4 recorded before (Pre) and after UTMC stimulation (Post-UTMC). Right: Corresponding changes in signal quantified as ΔRFU (Post – Pre, *n* = 4 per group). ****P < 0.0001 for UTMC group vs. other groups. ^####^*P* < 0.0001 for TG + UTMC group vs. Control group. ^††^*P* < 0.01 for Ca^2+^-free + UTMC group vs. Control group. (**H**) NOx concentration in culture supernatants following the indicated treatments and 3 UTMC cycles (*n* = 4 per group). ****P < 0.0001 for UTMC group vs. other groups. ^#^*P* < 0.05 for UTMC + BAPTA-AM group vs. other groups’ expected UTMC. Statistical analyses were performed using 1-way ANOVA followed by Tukey’s post hoc test. NS, no significance.

Repeated UTMC exposure at 3 ultrasound intensities significantly increased nitrate and nitrite (NOx) production in HUVECs (Figure 3A). In parallel, ATP release and adrenomedullin (ADM) secretion were progressively elevated with increasing UTMC cycles (Figure 3, B and C). Among the tested conditions, an ultrasound intensity of 25 mW/cm^2^ elicited the greatest responses, with NOx levels significantly higher than those observed in the 50 and 100 mW/cm^2^ groups after 3 UTMC cycles. Consistent with this trend, ATP and ADM levels were also markedly increased in the 25 mW/cm^2^ group compared with higher-intensity UTMC conditions, whereas no significant difference was detected between the 50 and 100 mW/cm^2^ groups across all cycles. These findings identify 25 mW/cm^2^ as the condition associated with the highest levels of NOx production and endothelial mediator release among the tested parameters.

Given previous reports that ATP and ADM regulate eNOS activation through distinct G protein–coupled signaling pathways (17), we assessed phosphorylation of eNOS at Ser1177 and Ser633 under different UTMC conditions. As shown in Figure 3, D and E, phosphorylation at both sites increased progressively with repeated UTMC exposure in HUVECs, with the strongest responses observed at 25 mW/cm^2^. These data demonstrate enhanced eNOS phosphorylation following UTMC exposure and are consistent with activation of G protein–associated signaling pathways.

### UTMC elevates intracellular Ca^2+^ levels and regulates eNOS-dependent NOx production in HUVECs.

UTMC treatment induced a marked increase in intracellular free Ca^2+^ concentration in HUVECs (Figure 3F). Repeated UTMC exposure under 3 ultrasound intensities significantly elevated intracellular Ca^2+^ levels compared with control groups (PBS, ultrasound alone, and microbubbles alone). The most pronounced responses were observed at an ultrasound intensity of 25 mW/cm^2^, where intracellular Ca^2+^ levels were significantly higher than in all control groups after 3 UTMC cycles. In contrast, higher ultrasound intensities (50 and 100 mW/cm^2^) elicited weaker increases in intracellular Ca^2+^, and no significant difference was observed between the 2 groups. These results identify 25 mW/cm² as the condition associated with the greatest cytosolic Ca^2+^ elevation among the tested parameters.

To determine the source of the UTMC-induced increase in cytosolic Ca^2+^, the relative contributions of extracellular Ca^2+^ influx and endoplasmic reticulum (ER) Ca^2+^ release were assessed using Fluo-4 fluorescence following pretreatment with Ca^2+^-free buffer supplemented with EGTA or thapsigargin (TG). As shown in Figure 3G, UTMC markedly increased intracellular Ca^2+^ levels compared with control. Depletion of ER Ca^2+^ stores with TG significantly attenuated, but did not abolish, the UTMC-induced Ca^2+^ rise. In contrast, removal of extracellular Ca^2+^ produced a more pronounced reduction in Fluo-4 fluorescence intensity. Quantitative analysis of the change in relative fluorescence units (ΔRFU; posttreatment minus pretreatment fluorescence intensity) indicated that extracellular Ca^2+^ removal accounted for the majority of the UTMC-evoked Ca^2+^ signal, whereas ER depletion contributed a smaller fraction. Under Ca^2+^-free conditions, TG further diminished the residual signal, supporting a secondary contribution from ER Ca^2+^ release. Neither TG nor Ca^2+^-free treatment alone significantly altered basal Ca^2+^ levels. Together, these findings indicate that extracellular Ca^2+^ influx represents the primary component of the UTMC-induced Ca^2+^ response, with ER-derived Ca^2+^ contributing to a lesser extent.

Given the established role of intracellular Ca^2+^ levels in eNOS mechanosensing and signaling, we next examined whether UTMC-induced elevation of intracellular Ca^2+^ levels contributes to NOx production. As shown in Figure 3H, buffering intracellular Ca^2+^ with BAPTA-AM markedly reduced UTMC-induced NOx production, and inhibition of eNOS with L-NAME nearly abolished NOx generation. These data support a model in which intracellular Ca^2+^ elevation contributes to UTMC-mediated eNOS-dependent NO production.

### UTMC-induced eNOS activation is associated with GNAS and GNAQ/GNA11 signaling in HUVECs.

**Figure 4:**
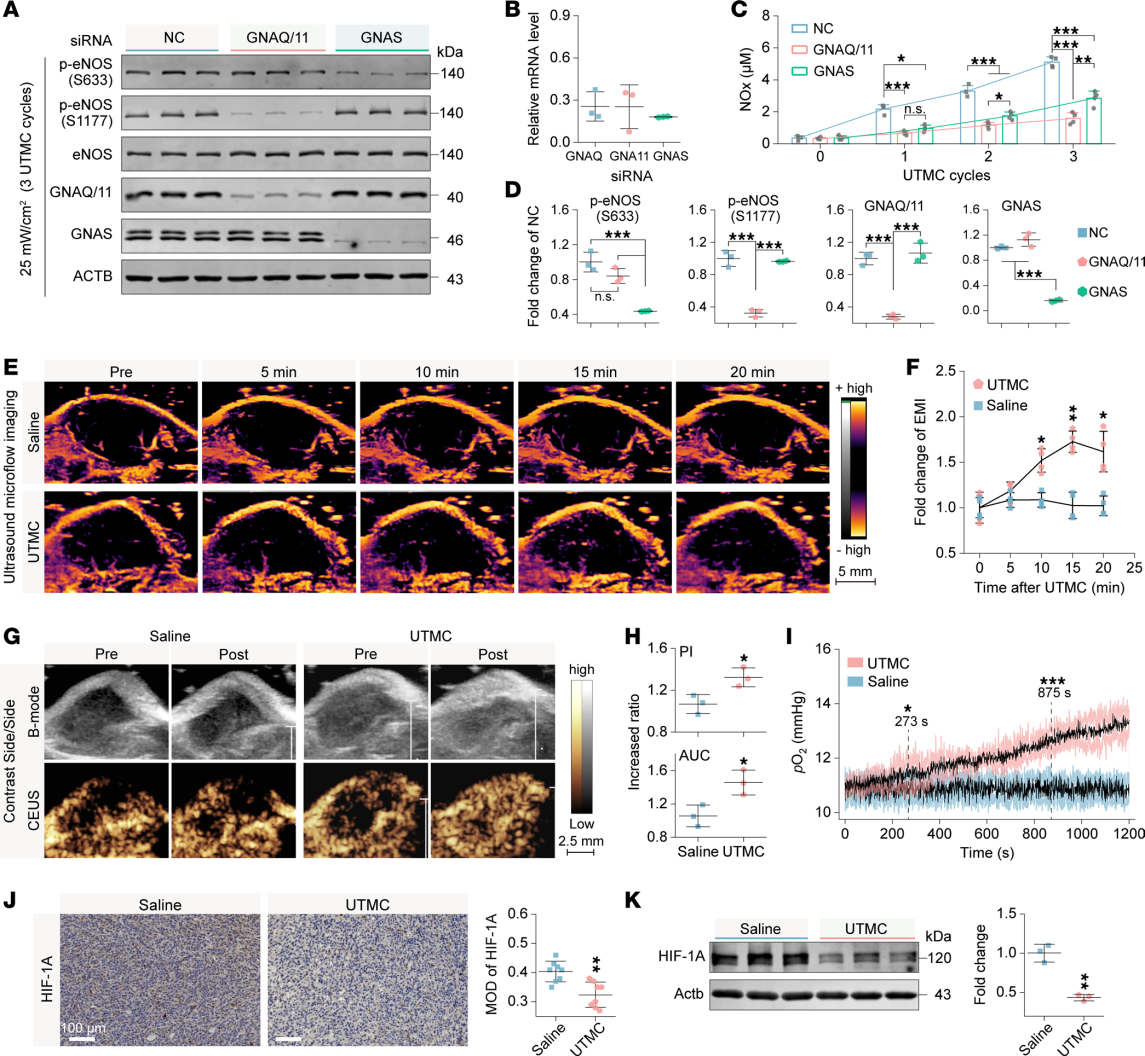
Effects of G protein knockdown on eNOS activation and tumor perfusion improvement by UTMC. (**A** and **D**) Western blot bands and corresponding quantitative analysis of siRNA-mediated G protein knockdown and the subsequent phosphorylation status of eNOS (*n* = 3 per group). NC, negative control. (**B**) Relative mRNA expression levels after siRNA transfection (*n* = 3 per group). (**C**) Measurement of NOx concentrations in the culture supernatants following G protein knockdown (*n* = 4 per group). (**E** and **F**) Representative ultrasound microflow imaging (MFI) of tumors in 4T1 tumor–bearing mice and corresponding temporal curves of EMI from 4 independent experiments. (**G**) Representative B-mode and CEUS images of the same tumors before and after treatment from 3 independent experiments. (**H**) Quantification of the relative increase in peak intensity (PI) and AUC of the same tumor after treatment, calculated as posttreatment/pretreatment (*n* = 3 per group). (**I**) *p*O_2_ measurement of tumor tissues for 20 minutes after treatment (*n* = 3 per group). Shaded regions indicate SD, and bold lines represent mean values. The first statistically significant difference between groups was observed at approximately 273 seconds, and group differences remained significant thereafter from approximately 875 seconds onward. (**J**) Representative immunohistochemical images of HIF-1A and corresponding quantitative analysis of mean optical density (MOD) in tumor tissues harvested 12 hours after treatment (scale bars: 100 μm). Representative images from 4 independent tumors per group are shown, with 2 tissue section analyzed from each tumor. (**K**) Western blot bands and corresponding quantitative analysis for HIF-1A in tumor tissues harvested 12 hours after treatment (*n* = 3 per group). The same samples were run in a separate gel for Western blotting. Statistical analyses were performed using 1-way ANOVA followed by Tukey’s post hoc test (**C** and **D**), 2-way ANOVA followed by Šidák’s post hoc test (**F** and **I**), or unpaired 2-tailed Student’s *t* test (**H**, **J**, and **K**). NS, no significance. *P < 0.05, **P < 0.01, ***P < 0.001.

Before assessing the roles of GNAQ/GNA11 (GNAQ/11) and GNAS in UTMC-mediated eNOS activation, siRNA-mediated knockdown of GNAQ/11 and GNAS was performed. Both quantitative reverse transcription PCR (qRT-PCR) and Western blot analyses confirmed efficient depletion of GNAQ/11 and GNAS (Figure 4, A, B, and D). Knockdown of GNAS did not affect UTMC-induced eNOS phosphorylation at Ser1177 but significantly reduced phosphorylation at Ser633. In contrast, knockdown of GNAQ/11 markedly decreased UTMC-induced eNOS phosphorylation at Ser1177, while also slightly reducing phosphorylation at Ser633. However, this reduction was not statistically significant compared with the negative control (Figure 4, A and D). Additionally, compared with the negative control group, NOx release was significantly reduced in both GNAQ/11-knockdown (0.31 ± 0.07-fold) and GNAS-knockdown (0.55 ± 0.08-fold) groups after 3 cycles of UTMC treatment, with a more pronounced reduction observed following GNAQ/11 knockdown (Figure 4C). Together, these findings demonstrate that both GNAQ/11 and GNAS contribute to UTMC-induced eNOS phosphorylation and NOx production, with GNAQ/11 knockdown exerting a stronger inhibitory effect under the tested conditions.

### UTMC enhances tumor perfusion and alleviates hypoxia in murine TNBC models.

Following local UTMC treatment of tumors, ultrasound microflow imaging (MFI) showed a gradual increase in EMI signals within the tumor region compared with saline controls (Figure 4E). Quantitative analysis revealed that EMI gradually increased after UTMC and peaked at approximately 15 minutes after treatment, reaching nearly 1.7-fold higher than baseline levels, whereas saline-treated tumors showed minimal changes over time (Figure 4F). Consistent with improved perfusion, CEUS imaging revealed enhanced intratumoral blood flow after UTMC, as reflected by significant increases in peak intensity and area under the curve (AUC) compared with saline-treated tumors (Figure 4, G and H).

To directly evaluate intratumoral oxygenation, real-time monitoring of tumor oxygen partial pressure (*p*O_2_) was performed. UTMC treatment induced a sustained elevation in intratumoral *p*O_2_ beginning within several minutes after treatment and persisting throughout the observation period, whereas saline-treated tumors showed no appreciable change (Figure 4I). Consistent with improved oxygenation, immunohistochemical analysis demonstrated a significant reduction in hypoxia-inducible factor-1α (HIF-1A) expression in UTMC-treated tumors compared with controls (Figure 4J). Western blot analysis further confirmed decreased HIF-1A protein levels following UTMC treatment (Figure 4K). Together, these findings demonstrate that UTMC treatment is associated with enhanced tumor microvascular perfusion and sustained improvement in intratumoral oxygenation.

### UTMC combined with anti–PD-L1 therapy enhances tumor growth inhibition in murine TNBC models.

**Figure 5:**
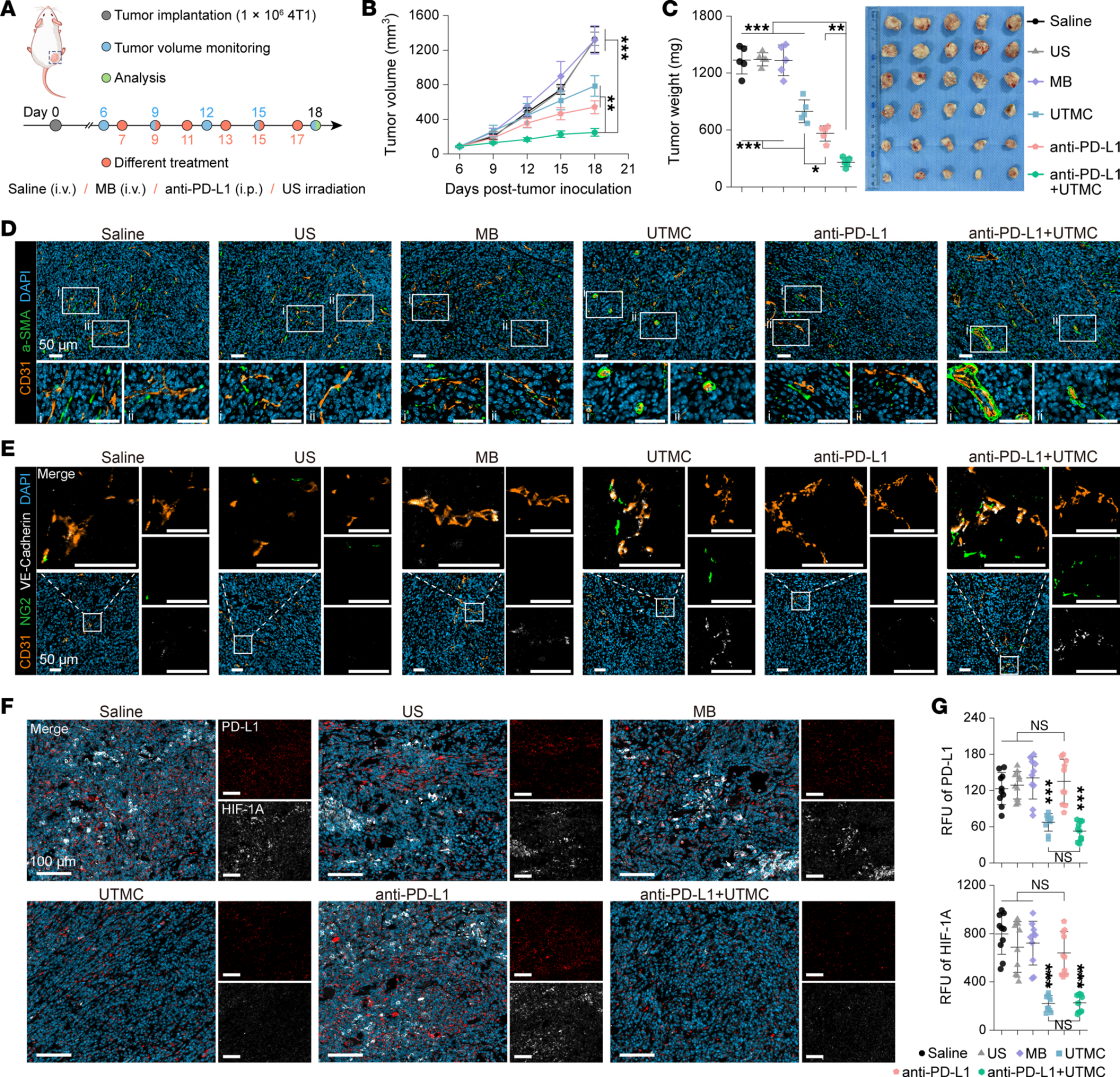
Evaluation of antitumor effects of UTMC combined with immune checkpoint blockade. (**A**) Schematic illustration of the monitoring and treatment protocol for 4T1 tumor–bearing mice. MB, microbubbles-alone control; US, ultrasound-alone control. (**B**) Average tumor growth curves in mice subjected to different treatments (*n* = 5 per group). (**C**) Tumor weights and corresponding digital images of tumors harvested on day 18 after treatment (*n* = 5 per group). (**D**) Representative multiplex immunofluorescent staining images for CD31, α-SMA, and DAPI in tumor tissues collected on day 18 following the indicated treatments. Representative images from 5 independent tumors per group are shown, with 1 tissue section analyzed from each tumor. (**E**) Representative multiplex immunofluorescent staining images for CD31, NG2, VE-cadherin, and DAPI in tumor tissues collected on day 18 following the indicated treatments. Representative images from 5 independent tumors per group are shown, with 1 tissue section analyzed from each tumor. (**F** and **G**) Representative multiplex immunofluorescent staining images and corresponding quantitative analysis of RFU for PD-L1, HIF-1A, and DAPI in tumor tissues collected on day 18 following the indicated treatments (blue indicates DAPI nuclear staining). Scale bars: 50 μm (**D**–**F**). Representative images from 5 independent tumors per group are shown, with 2 tissue sections analyzed from each tumor. Statistical analyses were conducted using 2-way ANOVA followed by Šidák’s post hoc test (**B**) or 1-way ANOVA followed by Tukey’s post hoc test (**C** and **G**). NS, no significance. *P < 0.05; **P < 0.01; ***P < 0.001.

Tumor growth curves and body weight changes in tumor-bearing mice were monitored throughout the treatment period (Figure 5A). No significant differences in body weight were observed among the groups, indicating an absence of apparent treatment-related toxicity (Supplemental Figure 2A). As shown in Figure 5B and Supplemental Figure 2B, UTMC alone, anti–PD-L1 monotherapy, or their combination inhibited tumor growth to varying extents. The combination of UTMC and anti–PD-L1 resulted in greater tumor growth inhibition compared with anti–PD-L1 monotherapy. Representative images of excised tumors and tumor weights measured on day 18 after treatment further supported these findings (Figure 5C).

### UTMC induces partial vascular normalization and reduces PD-L1 expression in murine TNBC tumors.

The effects of UTMC on tumor vasculature were assessed by multiplex immunofluorescent staining of CD31 and α-smooth muscle actin (α-SMA) (Figure 5D). CD31 expression showed no apparent differences among treatment groups. However, in tumors treated with UTMC or UTMC combined with anti–PD-L1, α-SMA exhibited prominent coverage surrounding CD31-positive vessels, whereas no notable changes in α-SMA distribution were observed with anti–PD-L1 treatment alone, suggesting enhanced mural cell association (include pericytes and vascular smooth muscle cells) and vascular stabilization (Figure 4E). To further evaluate vascular normalization features, multiplex immunofluorescent staining of CD31 together with NG2 and VE-cadherin was performed (Figure 5E). Increased NG2 and VE-cadherin signals were observed surrounding CD31-positive vessels in UTMC and UTMC plus anti–PD-L1 groups compared with other groups, indicating enhanced perivascular support and improved endothelial junction integrity. Additionally, multiplex immunofluorescent staining for HIF-1A and PD-L1 revealed significantly reduced expression of both markers in tumors from UTMC and UTMC plus anti–PD-L1 groups compared with anti–PD-L1 monotherapy (Figure 5, F and G). Together, these results indicate that UTMC promotes partial vascular normalization, alleviates tumor hypoxia, and is associated with reduced PD-L1 expression within the tumor.

### UTMC combined with anti–PD-L1 therapy activates antitumor immunity in murine TNBC models.

**Figure 6:**
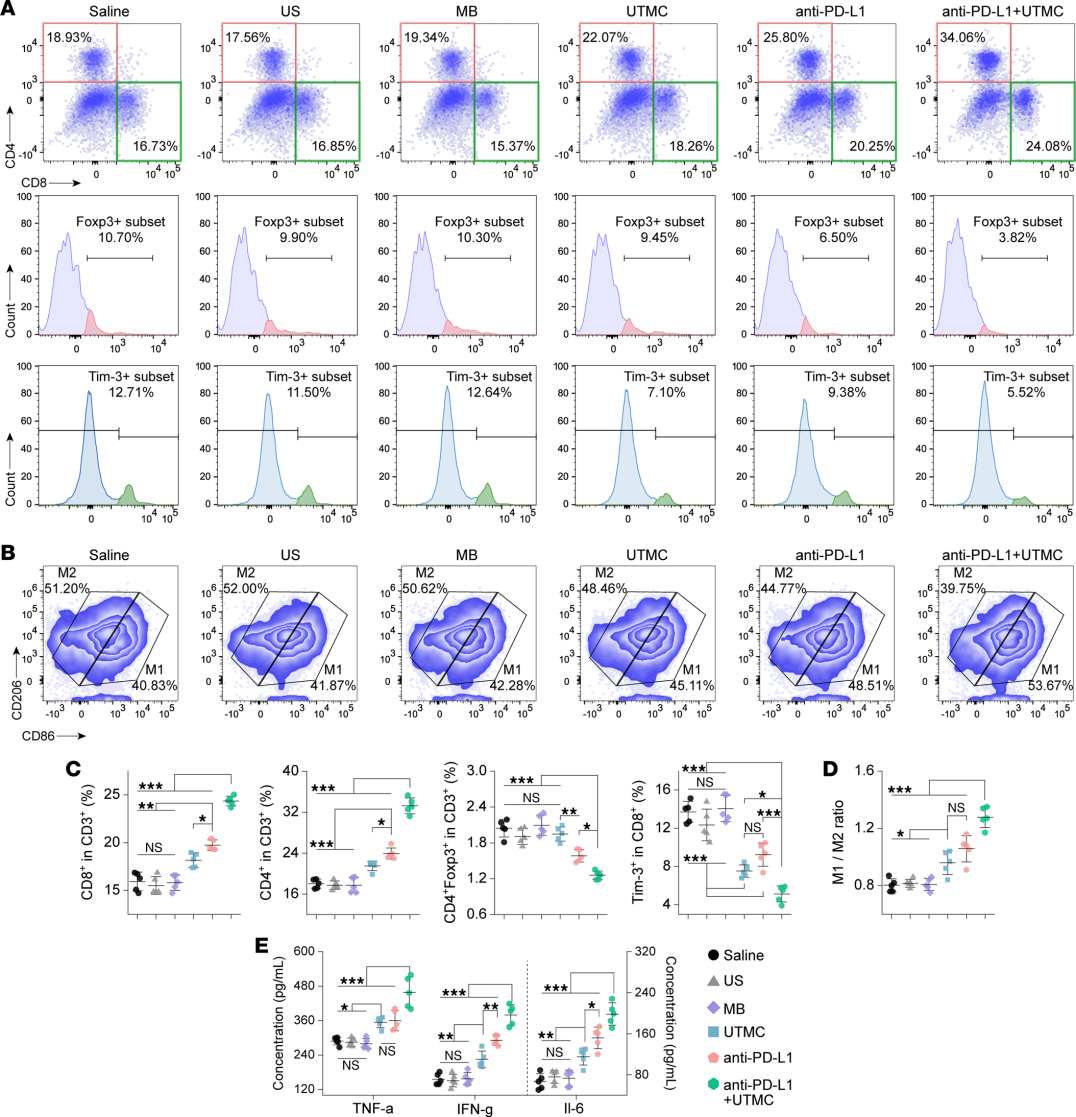
Evaluation of immune cell infiltration in tumor tissues following combined treatment. (**A** and **C**) Representative flow cytometry plots and corresponding quantitative analysis of intratumoral infiltration of CD4^+^ T cells (CD3^+^CD4^+^), CD8^+^ T cells (CD3^+^CD8^+^ Tim-3^+/–^), and Tregs (CD3^+^CD4^+^Foxp3^+^) in tumor tissues collected on day 18 following the indicated treatments. Representative flow cytometry plots from 5 independent tumors per group are shown. MB, microbubbles-alone control; US, ultrasound-alone control. (**B** and **D**) Representative flow cytometry plots and corresponding quantitative analysis of intratumoral infiltration of M1 (CD11b^+^F4/80^+^CD86^hi^CD206^lo^) and M2 (CD11b^+^F4/80^+^CD86^lo^CD206^hi^) macrophages in tumor tissues collected on day 18 following the indicated treatments. Representative flow cytometry plots from 5 independent tumors per group are shown. (**E**) Quantitative measurements of TNF-α, IFN-γ, and IL-6 concentrations in tumor tissues harvested on day 18 after treatment (*n* = 5 per group). Statistical analyses were conducted using 1-way ANOVA followed by Tukey’s post hoc test. NS, no significance. *P < 0.05; **P < 0.01; ***P < 0.001.

As shown in Figure 6, A–D, flow cytometry analyses demonstrated that treatment with UTMC, anti–PD-L1 monotherapy, or their combination significantly increased intratumoral infiltration of CD8^+^ and CD4^+^ T cells and elevated the M1/M2 macrophage ratio. In addition, anti–PD-L1 alone and combined with UTMC markedly reduced intratumoral Treg proportions. UTMC or anti–PD-L1 monotherapy significantly decreased Tim-3 expression on CD8^+^ T cells, whereas combination therapy achieved a greater reduction, consistent with reduced T cell exhaustion.

**Figure 7:**
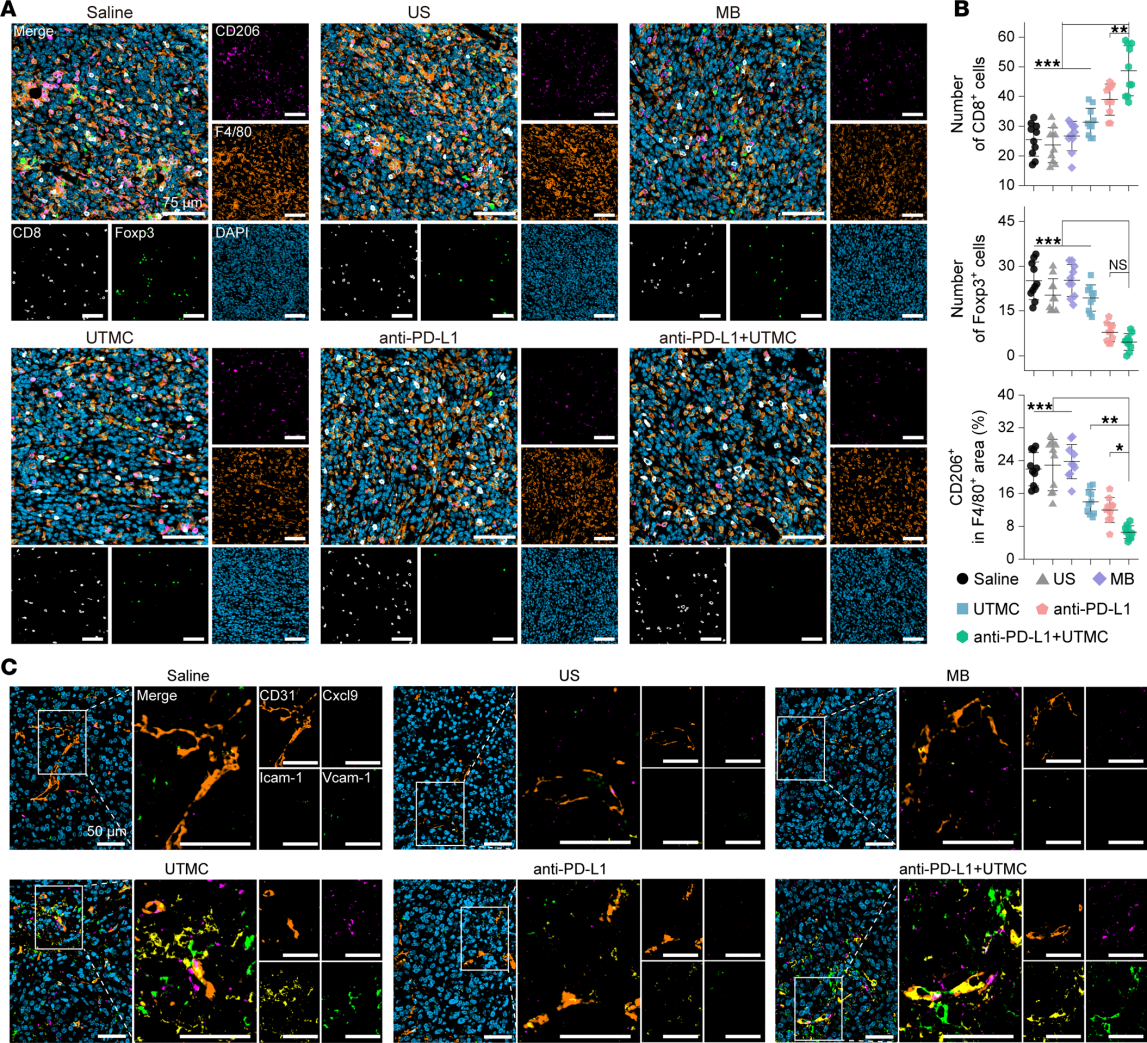
Multiplex immunofluorescence analysis of tumor tissues following UTMC combined with immune checkpoint blockade treatment. (**A** and **B**) Representative multiplex immunofluorescence images and corresponding quantitative analysis of immune cell infiltration in tumor tissues collected on day 18 after various treatments (scale bars: 75 μm). Representative images from 5 independent tumors per group are shown, with 2 tissue section analyzed from each tumor. MB, microbubbles-alone control; US, ultrasound-alone control. (**C**) Representative multiplex immunofluorescent staining images for CD31, Cxcl9, Icam-1, Vcam-1, and DAPI in tumor tissues collected on day 18 following the indicated treatments (blue indicates DAPI nuclear staining). Scale bars: 50 μm. Representative images from 5 independent tumors per group are shown, with 1 tissue section analyzed from each tumor. Statistical analyses were conducted using 1-way ANOVA followed by Tukey’s post hoc test. NS, no significance. *P < 0.05, **P < 0.01, ***P < 0.001.

Consistently, combined UTMC and anti–PD-L1 treatment induced significantly higher intratumoral levels of the proinflammatory cytokines TNF-α, IFN-γ, and IL-6 (Figure 6E). Multiplex immunohistochemistry further confirmed enhanced CD8^+^ T cell infiltration following combination therapy compared with other treatment groups, while anti–PD-L1 monotherapy and combination treatment both reduced intratumoral Treg infiltration. In addition, UTMC, anti–PD-L1, and their combination significantly decreased the proportion of M2 macrophages (Figure 7, A and B).

To investigate potential mechanisms underlying increased immune infiltration, multiplex immunofluorescent staining was performed to assess vasculature-associated trafficking signals within the TME. As shown in Figure 7C, expression of the endothelial adhesion molecules Icam-1 and Vcam-1, together with the T cell–recruiting chemokine Cxcl9, was markedly increased in tumors treated with UTMC compared with saline, ultrasound, or microbubble controls and was predominantly localized around CD31-positive vasculature. Notably, combined UTMC and anti–PD-L1 treatment further enhanced Icam-1, Vcam-1, and Cxcl9 expression relative to anti–PD-L1 monotherapy, consistent with enhanced vasculature-associated immune cell recruitment signals. Together, these findings demonstrate that UTMC, particularly in combination with anti–PD-L1 therapy, is associated with increased effector T cell infiltration, reduced T cell exhaustion, decreased immunosuppressive cell populations, and enhanced proinflammatory cytokine production within the TME.

## Discussion

TNBC is characterized by limited therapeutic options and poor prognosis, largely due to its inherent heterogeneity and resistance to conventional treatments. ICB therapy has recently emerged as a promising treatment strategy for TNBC, yet durable clinical benefit is observed only in a subset of patients. Tumor hypoxia has been increasingly recognized as a critical contributor to immune evasion, therapeutic resistance, and aggressive disease progression (4, 5), highlighting hypoxia alleviation as a potentially valuable adjunct to enhance the efficacy of immunotherapy.

In this study, we integrated transcriptomic cohorts from TCGA and GEO to examine the immunological landscape of TNBC stratified by hypoxia status. By stratifying TNBC according to a HRG signature, we observed that tumors categorized as high-hypoxia exhibited markedly elevated expression of multiple ICGs compared with low-hypoxia tumors. Specifically, several key ICGs, including CD276 (a known inhibitor of T cell activation and proliferation, ref. 20), CD274 (also known as PD-L1), PDCD1 (also known as PD-1), and CD44 (previously associated with metastasis, recurrence, and drug resistance; ref. 21), were markedly upregulated in the high-hypoxia group. The coordinated upregulation of these immune checkpoint molecules supports a hypoxia-associated immunosuppressive phenotype characterized by impaired cytotoxic T cell activity and enhanced co-inhibitory signaling.

Immune-related signature analysis revealed increased infiltration of MDSCs and TAMs in high-hypoxia tumors, associated with immunosuppression and poor clinical outcomes in patients with cancer (22). High-hypoxia tumors also exhibited elevated T cell exhaustion signatures and increased expression of exhaustion-associated markers such as HAVCR2 (Tim-3) (23, 24). Consistent with these findings, TIDE analysis predicted reduced responsiveness to ICB in the high-hypoxia group, accompanied by increased T cell dysfunction scores.

Notably, we observed higher T cell–inflamed GEPs in the high-hypoxia group compared with the low-hypoxia group. Previous studies have demonstrated that elevated T cell–inflamed GEP scores typically predict better responsiveness to PD-1/PD-L1 ICB therapies, as they reflect enhanced T cell infiltration and IFN-γ signaling within the TME (25). However, tumors with higher T cell–inflamed GEP scores also often exhibit concurrent immune escape mechanisms, characterized by increased expression of immunosuppressive checkpoint molecules. Together, these observations highlight a complex interplay within the hypoxic TNBC TME. High hypoxia is therefore associated with an immune-infiltrated yet functionally suppressed state, characterized by increased immunosuppressive myeloid cell infiltration, exhausted T cell populations, and reduced predicted responsiveness to ICB. These findings provide a rationale for combining hypoxia-modulating strategies with ICB in TNBC. Notably, hypoxia status was not an independent prognostic factor in survival analyses, suggesting that modulation of hypoxia alone may be insufficient to alter clinical outcomes in the absence of concurrent immune activation.

In recent years, UTMC has emerged as a mechanistically grounded method to enhance vasodilation and tumor perfusion, informed primarily by findings from fluid shear stress studies. Fluid shear stress generated by blood flow stimulates endothelial cells to release NO, a potent vasodilator, via signaling pathways involving mechanosensors such as Piezo1 and downstream mediators, including ADM/GNAS/PKA and ATP/GNAQ/GNA11/PDK1/AKT (17–19). These pathways trigger phosphorylation of eNOS at multiple regulatory sites, thereby enhancing NO production and promoting vasodilation. However, given the differences in mechanical stimuli between UTMC and conventional fluid shear stress (7, 26, 27), the precise mechanotransduction pathways activated by UTMC remain unclear and require direct validation.

Before exploring these mechanisms, we evaluated whether UTMC induces endothelial cell injury. We observed a dose-dependent decrease in endothelial cell viability and a corresponding increase in PI positivity with rising microbubble concentrations and ultrasound intensities, indicating mechanical injury. Notably, even under high-intensity conditions (e.g., microbubbles diluted 10-fold with an ultrasound intensity of 400 mW/cm^2^ and microbubbles diluted 20-fold with an ultrasound intensity of 1000 mW/cm^2^), cell viability consistently remained above 80%, despite significant membrane disruption (PI positivity > 20%). These findings suggest that UTMC can modulate endothelial permeability while preserving overall cell viability, a property relevant to its translational application (28, 29).

We further provide mechanistic evidence that UTMC-induced eNOS activation involves coordinated G protein–associated signaling through GNAS and GNAQ/11 pathways. Following UTMC stimulation, endothelial cells exhibited substantially elevated release of ATP and ADM, two key mediators previously established as initial signaling factors in fluid shear stress–induced vasodilation pathways. ATP was rapidly released during the early phase of UTMC exposure, whereas ADM levels markedly increased at later stages, indicating a coordinated temporal regulation of these pathways. Previous studies demonstrated that short-term fluid shear stress–induced NO production predominantly relies on GNAQ/11-mediated signaling, while sustained shear stress activates both GNAQ/11 and GNAS signaling pathways (18). In parallel with these models, our data suggest that ATP-associated signaling predominates during early UTMC exposure, whereas ADM-associated signaling contributes during repeated stimulation cycles. Together, these findings support a model in which UTMC engages temporally coordinated G protein–associated signaling pathways that partially overlap with classical shear stress mechanisms.

Knockdown experiments demonstrated differential regulation of eNOS phosphorylation, with GNAQ/11 primarily affecting Ser1177 and GNAS preferentially influencing Ser633. Both phosphorylation events contributed to NOx production following UTMC stimulation. In contrast with previous reports in shear stress models showing reduced phosphorylation at both Ser633 and Ser1177 in GNAS-deficient settings (17), we did not observe a significant reduction in Ser1177 phosphorylation following GNAS knockdown. This divergence may reflect differences in mechanical force dynamics and suggests that UTMC could engage additional mechanosensitive pathways, including Pecam1/VE-cadherin/PI3K/AKT signaling (30–33).

Importantly, we further delineated the upstream calcium dependency of UTMC-induced eNOS activation. Using extracellular Ca^2+^ depletion and TG-mediated ER store depletion, we demonstrated that extracellular Ca^2+^ influx constituted the dominant component of the UTMC-evoked Ca^2+^ response, with ER-derived Ca^2+^ contributing secondarily. Buffering intracellular Ca^2+^ with BAPTA-AM markedly attenuated NOx production, whereas pharmacologic inhibition of eNOS with L-NAME nearly abolished NO generation. These complementary approaches establish a Ca^2+^-dependent, eNOS-mediated mechanism linking UTMC-induced mechanical stimulation to NO production.

In this study, UTMC monotherapy markedly inhibited tumor growth, a response associated with improved tumor perfusion and remodeling of the tumor immune microenvironment. Previous studies have shown that hypoxia promotes breast cancer cell growth by activating a glycogen metabolic program (34). Thus, improved tumor perfusion and alleviation of hypoxia may disrupt hypoxia-associated metabolic advantages. In addition to these vascular effects, UTMC was associated with increased CD8^+^ T cell infiltration, an elevated M1/M2 macrophage ratio, reduced T cell exhaustion, and increased proinflammatory cytokine levels. Together, these findings indicate that UTMC shifts the TME from an immunosuppressive toward an immune-activated state, thereby strengthening endogenous antitumor immunity and ultimately suppressing tumor growth.

Consistent with mechanistic findings, in vivo analyses demonstrated improved perfusion and reduced HIF-1A expression following UTMC. This improved perfusion was accompanied by increased pericyte coverage, as shown by elevated α-SMA expression surrounding endothelial cells, suggesting vascular normalization rather than angiogenesis as the predominant mechanism (35, 36). Importantly, multiplex immunofluorescence further demonstrated increased NG2 and VE-cadherin signals surrounding CD31-positive vessels in UTMC-treated tumors, indicating enhanced perivascular support and improved endothelial junction integrity. These structural features are hallmarks of partial vascular normalization and support the conclusion that UTMC stabilizes tumor vasculature. Vascular normalization is known to restore microenvironmental homeostasis and facilitate immune cell infiltration (37), thereby creating conditions more permissive for effective antitumor immunity.

To further explore how UTMC facilitates immune cell recruitment, we assessed vasculature-associated trafficking signals within the TME. UTMC markedly increased the expression of the endothelial adhesion molecules Icam-1 and Vcam-1, as well as the T cell–recruiting chemokine Cxcl9, predominantly localized around CD31-positive vasculature. Combined UTMC and anti–PD-L1 treatment further enhanced these signals compared with anti–PD-L1 monotherapy, suggesting that UTMC promotes a vascular microenvironment permissive for immune cell adhesion and transmigration.

Additionally, UTMC markedly decreased PD-L1 expression within tumors, further attenuating immunosuppressive signaling and potentially augmenting antitumor immunity. Notably, combining UTMC with anti–PD-L1 therapy substantially suppressed tumor growth compared with anti–PD-L1 monotherapy. This combined treatment markedly increased infiltration of cytotoxic CD8^+^ T cells, reduced T cell exhaustion, reduced Treg populations, and elevated proinflammatory cytokine levels. Furthermore, UTMC treatment decreased macrophage polarization toward the immunosuppressive M2 phenotype, shifting the balance toward an immunostimulatory M1-dominant profile.

Collectively, these findings indicate that UTMC remodels the tumor vasculature and immune microenvironment through coordinated vascular stabilization, enhancement of endothelial immune-trafficking signals, downregulation of PD-L1 expression, and macrophage reprogramming toward an M1-dominant phenotype. These integrated effects provide mechanistic support for UTMC as a rational adjunctive strategy to improve responsiveness to ICB in TNBC.

Although UTMC-induced reoxygenation was associated with reduced tumor burden and immune activation, reoxygenation may also entail adverse consequences. Oxygen influences fibroblast activation, collagen deposition, angiogenesis, and epithelialization (38), and may under certain conditions promote tumor progression. Emerging evidence suggests that reoxygenation may also affect tumor heterogeneity and adaptive resistance. Importantly, in this study, reoxygenation occurred concurrently with immune activation and vascular normalization rather than as an isolated oxygen increase, potentially mitigating protumorigenic effects. These considerations represent a limitation and warrant further investigation.

## Methods

### Sex as a biological variable.

Our study exclusively examined female mice because the disease modeled is only relevant in females.

### Public data acquisition.

The TNBC cohort was retrieved from TCGA (https://portal.gdc.cancer.gov/) using the R software (version 4.5.0; https://www.r-project.org/) package TCGAbiolinks (version 2.25.3). After excluding samples lacking clinical annotations, RNA-seq data (in counts format) and corresponding clinical information from 122 TNBC samples were included in the analysis. RNA-seq data were subsequently normalized into transcripts per million (TPM) and log_2_-transformed prior to analysis. Clinical annotations were obtained from the UCSC Xena database (https://xena.ucsc.edu/). Additionally, transcriptomic microarray data (GSE76275) of TNBC were downloaded from the NCBI GEO database. Raw data in CEL format were normalized using the robust multi-array average method implemented in the R package oligo (version 1.70.2). Probe annotations were obtained from the hgu133plus2.db R package (version 3.13.0), and duplicate probes for the same gene were averaged.

### Hypoxia-based stratification of TNBC in TCGA and GEO cohorts.

HRGs were acquired from the GeneCards database (https://www.genecards.org/) by searching with the keyword “Hypoxia,” filtering for protein-coding genes, and selecting those with relevance scores exceeding the median. Ultimately, a total of 3,095 HRGs were identified and included in subsequent analyses. For each cohort, hypoxia scores were calculated using ssGSEA implemented in the GSVA R package (version 2.2.0) with default parameters. The ssGSEA enrichment score was defined as the hypoxia score for each sample. Within each cohort, patients were stratified into high- and low-hypoxia groups based on the cohort-specific median hypoxia score. This median-based dichotomization was used to avoid arbitrary cutoff selection and to ensure balanced group sizes for downstream comparative analyses.

### Immune signature scoring and immune checkpoint analysis in TCGA and GEO cohorts.

Immune-related functional signatures were evaluated using the R package IOBR (version 0.99.0) (39–43). Signature scores reflecting immune checkpoint activity, T cell exhaustion, Treg abundance, CAF abundance, EMT, ICB resistance, MDSCs, TAMs, TGFB1 family member and receptor signaling, and Pan-F-TBRs were calculated using the “calculate_sig_score()” function with the ssGSEA method and default parameters in IOBR. Additionally, a panel of 45 ICGs was identified from previously published literature from PubMed (https://pubmed.ncbi.nlm.nih.gov/) and incorporated into subsequent analyses (44, 45). The resulting functional scores were compared between TNBC patients stratified into high- and low-hypoxia groups based on hypoxia scores, and visualized as heatmaps generated using the R package ComplexHeatmap (version 2.18.0).

### T cell exhaustion scores and T cell–inflamed gene expression profile in TNBC from TCGA and GEO cohorts.

Wherry T cell exhaustion scores (46, 47) and the T cell–inflamed GEPs (25) were independently calculated using *Z*-score normalization and ssGSEA. *Z*-scores were obtained by standardizing the expression values of the relevant gene signatures across samples. ssGSEA scores were computed with the R package GSVA (version 2.2.0).

### TIDE analysis for immunotherapy response prediction in TNBC from the TCGA-TNBC cohort.

RNA-seq data from TCGA-TNBC samples were normalized by log_2_(TPM + 1) according to the requirements specified by the TIDE analysis platform (http://tide.dfci.harvard.edu/). The normalized expression data were subsequently uploaded to the TIDE web server. Prediction scores, including TIDE, T cell dysfunction, and T cell exclusion, were retrieved and imported into R for further statistical analyses.

### Ultrasonic machines and microbubbles.

The low-intensity pulsed ultrasound (LIPUS) LIPU-STIM320 therapeutic system, equipped with a planar therapeutic transducer (area, 4 cm^2^; frequency, 1 MHz; duty cycle, 10%; pulse repetition frequency, 100 Hz), was obtained from SXUltrasonic. An EPIQ 5 ultrasound system equipped with an eL18-4 transducer (Philips Healthcare) and SonoVue microbubble contrast agent (Bracco SPA) were provided by the Department of Ultrasound at First Affiliated Hospital of Harbin Medical University. The standard microbubble suspension was prepared according to the manufacturer’s instructions by reconstituting the lyophilized SonoVue microbubbles with 5 mL sterile saline to obtain the stock working solution. For in vivo and in vitro experiments, microbubble working solutions were prepared by further diluting this stock suspension with sterile saline to the indicated concentrations. Dilution factors are expressed relative to the reconstituted stock solution; for example, “100×” denotes a 100-fold dilution of the standard working suspension. To preserve microbubble integrity, all dilutions were performed using a sterile 3-way connector to allow gentle and controlled mixing, rather than repeated pipetting or dilution in microcentrifuge tubes, thereby minimizing shear stress and bubble disruption. The prepared suspensions were used immediately after dilution to reduce microbubble loss.

### Cells and animals.

HUVECs were purchased from Zhong Qiao Xin Zhou Biotechnology Co., Ltd. (ZQ1009) and cultured in specialized endothelial cell medium (1001, ScienCell). 4T1 cells were obtained from Procell Life Science & Technology Co., Ltd. (CL-0007) and cultured in RPMI-1640 complete medium (CM-0007, Procell). All cells were maintained at 37°C in a humidified atmosphere containing 5% CO_2_. BALB/c female mice (6 weeks old) were purchased from Changsheng Biotechnology Co., Ltd. All mice in this study were anesthetized using the same protocol. Specifically, anesthesia was induced by i.p. injection of tribromoethanol (2.5% Avertin, 100 μL per 10 g body weight) at a consistent dosage across all groups. No additional sedatives or supportive agents were administered.

### Fabrication of agarose phantom and optimization experiments.

Agarose powder was mixed with double-distilled water to prepare a 2% (w/v) solution at room temperature. The mixture was heated until all powder was fully dissolved and free of air bubbles. Subsequently, the agarose solution was poured into a specialized container and allowed to solidify before use in further experiments. A detailed workflow of the agarose phantom preparation is provided in Supplemental Figure 3.

To quantitatively evaluate the relationship between microbubble concentration and EMI, SonoVue microbubble suspensions with different dilution ratios were injected into the pores of the agarose phantom and observed using the CEUS mode of the EPIQ 5 ultrasound system. CEUS imaging was performed in C-Pen mode with the following settings: ultrasound gain at 45%, dynamic range at 55, persistence set to low, and mechanical index adjusted to 0.06. Images were captured, and EMI values of ROI were analyzed using PHILIPS QLAB software (version 13.0). The relationship between dilution ratios and corresponding EMI values was assessed by linear regression analysis.

To investigate the effects of LIPUS on SonoVue microbubbles, contrast agent solutions at various dilution ratios were individually injected into the pores of the agarose phantom and irradiated using a therapeutic ultrasound transducer. To minimize contrast agent loss during the dilution process, a 3-way stopcock system was used for controlled dilution. All subsequent experiments involving different dilution ratios of SonoVue microbubbles were prepared using the same 3-way stopcock–based dilution method to ensure consistency and reproducibility. Following irradiation for predetermined durations, microbubble behavior was observed using CEUS mode, and images were captured. The EMI values within selected ROIs were subsequently quantified using QLAB software.

### Evaluation of UTMC-induced cellular injury in HUVECs.

The effects of UTMC under various parameters on the viability and cell membrane integrity of HUVECs were evaluated using the Cell Counting Kit-8 (CCK-8) assay (CK04, Dojindo), PI staining, and Calcein AM/PI dual fluorescence staining (C2015S, Beyotime), respectively.

For viability assessment, HUVECs were seeded into 96-well plates and cultured until approximately 80% confluent. Subsequently, the culture medium in each well was replaced with microbubble contrast agent suspensions at various dilution ratios. Immediately following medium replacement, cells were exposed to continuous ultrasound irradiation for 5 minutes from the bottom of the plate using a therapeutic transducer. After irradiation, microbubble contrast agents were removed, and fresh serum-free culture medium was added. Cells were then incubated for an additional 16 hours at 37°C in an atmosphere containing 5% CO_2_, and cell viability was assessed using the CCK-8 assay.

To assess cell membrane integrity, HUVECs were seeded into 12-well plates and subjected to the same treatment procedure described above. After treatment and removal of microbubble contrast agents, cells were harvested by digestion with trypsin (without EDTA) to obtain single-cell suspensions. The harvested cells were stained with PI, and flow cytometry was performed to quantify the percentage of PI-positive cells.

To further visualize membrane integrity and cell viability, Calcein AM/PI dual fluorescence staining was performed. HUVECs were seeded into 12-well plates containing sterile glass coverslips and treated with UTMC under the indicated ultrasound intensities and microbubble dilution ratios. Following treatment, cells were gently washed with PBS and incubated with Calcein AM/PI working solutions according to the manufacturer’s instructions for 20 minutes at 37°C in the dark. After staining, cells were washed with PBS to remove excess dye, and fluorescence images were captured using a Leica TCS SP8 confocal laser scanning microscope.

### Evaluation of the mechanical effects of UTMC on eNOS activation in HUVECs.

HUVECs were seeded into 96- or 12-well plates and cultured until approximately 80% confluence was achieved. The culture medium in each well was then replaced with microbubble contrast agent (100× dilution), using 30 μL for 96-well plates and 200 μL for 12-well plates. Immediately following medium replacement, cells were irradiated for 1 minute from the bottom of the plate using a therapeutic ultrasound transducer, defined as 1 UTMC cycle. After each UTMC treatment cycle, the culture medium was promptly replaced with fresh medium containing microbubbles, and the next UTMC cycle was initiated under the same conditions. PBS, ultrasound alone, and microbubbles alone at the same concentration were used as control groups. The effects of 1 to 3 UTMC cycles on eNOS activation in HUVECs were evaluated separately.

To determine NOx, ADM, and ATP levels, cell supernatants in 12-well plates were collected after each UTMC cycle. Cellular debris and lipid components were removed by centrifugation at 20,000*g* for 10 minutes at 4°C. The concentrations of NOx, ADM, and ATP were then measured using a NOx Fluorometric Assay Kit (780051, Cayman Chemical), an ADM ELISA Kit (JL11786, Jianglai Biology), and an ATP Assay Kit (S0027, Beyotime), respectively, following the manufacturers’ instructions.

Phosphorylation of eNOS at Ser633 and Ser1177 in HUVECs cultured in 12-well plates was evaluated by Western blotting after different UTMC cycles. Detailed procedures for Western blotting are provided in the subsequent sections.

Intracellular Ca^2+^ concentrations were measured using Fluo-4 (S1061, Beyotime). HUVECs were seeded in 96-well black plates (FCP966, Beyotime) and preincubated with Fluo-4 prior to various treatments. After each UTMC treatment cycle, the culture medium was promptly replaced with fresh medium containing microbubbles, and the next UTMC cycle was initiated under the same conditions. RFU reflecting intracellular Ca^2+^ levels were quantified using a multimode microplate reader (Cytation 5, Agilent BioTek) after each UTMC. All incubation and fluorescence detection procedures were performed according to the manufacturer’s instructions. To determine the source of UTMC-induced Ca^2+^ elevation, cells were pretreated with Ca^2+^-free buffer supplemented with EGTA (a calcium chelator that binds extracellular Ca^2+^ and prevents Ca^2+^ influx) or with 1 μM TG (an inhibitor of sarco/endoplasmic reticulum Ca^2+^-ATPase that depletes ER Ca^2+^ stores by blocking Ca^2+^ reuptake into the ER) for 20 minutes. Following pretreatment, 3 cycles of UTMC stimulation were performed, and changes in Fluo-4 fluorescence were immediately recorded. Quantitative analysis was conducted by calculating ΔRFU (posttreatment minus pretreatment fluorescence intensity).

To assess the role of intracellular Ca^2+^ in UTMC-induced NO production, cells were pretreated with 50 μM BAPTA-AM (HY-100545, MedChemExpress; an intracellular Ca^2+^ chelator that buffers cytosolic Ca^2+^ and prevents Ca^2+^/calmodulin-dependent eNOS activation) for 20 minutes prior to UTMC stimulation. To confirm the enzymatic dependence of NO production, cells were pretreated with 200 μM L-NAME ((HY-18729, MedChemExpress; a competitive inhibitor of eNOS that inhibits the catalytic conversion of L-arginine to NO without directly affecting eNOS phosphorylation) for 20 minutes before UTMC exposure. Following 3 UTMC cycles, culture supernatants were collected and NOx levels were quantified as described above.

### Evalution of the effect of G protein knockdown on UTMC-induced eNOS activation in HUVECs.

To further elucidate the signaling pathways underlying UTMC-induced phosphorylation of eNOS at residues Ser633 and Ser1177, individual knockdown experiments targeting G proteins GNAS and GNAQ/11 were conducted using siRNA. Knockdown efficiency was verified by qRT-PCR and Western blotting, evaluating mRNA and protein expression levels of the targeted genes, respectively. Subsequently, HUVECs with confirmed knockdown of G proteins were exposed to UTMC (100× dilution, 25 mW/cm^2^). Following UTMC treatment, NOx concentrations in the culture supernatants and the phosphorylation status of eNOS residues in the cells were analyzed.

### siRNA-mediated knockdown and qRT-PCR analysis.

HUVECs were seeded in 6-well plates and cultured to approximately 60%–70% confluence. Transfection was performed using jetPRIME transfection reagent (Polyplus-transfection) according to the manufacturer’s instructions. Briefly, a siRNA-jetPRIME mixture (containing 110 pmol siRNA, 4 μL jetPRIME reagent, and 200 μL provided buffer) was prepared, incubated for 15 minutes at room temperature, and subsequently added to the medium. After 72 hours, total RNA was extracted using TRIzol reagent (15596018, Invitrogen), followed by reverse transcription with PrimeScript Reagent Kit with gDNA Eraser (RR047A, TaKaRa). qRT-PCR was performed using SYBR Green (Q204, NovaBio) on an Applied Biosystems StepOne. Relative gene expression levels were normalized to β-actin (ACTB), calculated by the 2^−ΔΔCt^ method, and expressed as fold changes compared to controls. Primer and siRNA sequences are provided in Supplemental Tables 2 and 3.

### Western blotting.

HUVECs seeded in a 12-well plate were treated under various conditions and then were lysed 2 times on ice using RIPA lysis buffer containing a mixture of protease inhibitor cocktails (05892970001 and 4906845001, Sigma-Aldrich) for 10 minutes. Normalized protein abundance was quantified by the bicinchoninic acid protein assay kit (P0012, Beyotime) and a BSA standard curve. Then, 20–60 μg of each sample was layered with SDS-polyacrylamide gels in SDS running buffer, and transferred to polyvinylidene fluoride membranes. After blocking with 5% nonfat dry milk at room temperature for 1 hour, primary antibodies against eNOS (1:1000; ab76198, Abcam), eNOS p-Ser1177 (1:1000; ab215717, Abcam), eNOS p-Ser633 (1:1000; sc-136198, Santa Cruz Biotechnology), GNAS (1:1000; ab283266, Abcam), GNAQ/11 (1:1000; sc-515689, Santa Cruz Biotechnology), HIF-1A (1:1000; ab179483, Abcam), and β-actin (Actb) (1:1000; 66009-1-Ig, Proteintech) were incubated with the membranes overnight. After washing with TBST, the membranes were incubated with fluorophore-specific secondary antibodies at room temperature for 1 hour to visualize the protein signals with a LI-COR Odyssey CLx Imaging System. LI-COR Image Studio Software was used to quantify the protein expression.

### Establishment of a unilateral subcutaneous tumor-bearing mouse model.

The tumor model was established by subcutaneously injecting 4T1 cells (1 × 10^6^ cells) into the right flank of 6-week-old BALB/c healthy female mice. After 6 days, when the tumor volume reached approximately 70−100 mm^3^ (tumor volume calculated using the formula, tumor volume = π/6 × length × width^2^), subsequent experiments were performed. Mice were humanely euthanized when tumor volumes exceeded 1,500 mm^3^ or ulcerations (>5 mm in diameter) developed.

### Evaluation of the effects of UTMC on alleviating hypoxia in murine TNBC models.

For tumor perfusion and hypoxia improvement experiments, mice were randomly divided into 2 groups: saline and UTMC. For administration, 200 μL of saline with/without MB (100×) was administered via tail-vein injection (i.v.). For ultrasound treatment, a small amount of ultrasound coupling gel was applied to the skin overlying the tumor, and the therapeutic transducer was placed directly over the tumor site as illustrated in Supplemental Figure 4. LIPUS was delivered locally to the tumor 5 seconds after microbubble injection and maintained continuously for 10 minutes. The acoustic parameters of the therapeutic transducer were 1 MHz, 10 % duty cycle, 100 Hz pulse repetition frequency, and intensity of 25 mW/cm^2^.

The perfusion level of the tumor was evaluated by MFI mode of the EPIQ 5 ultrasound system at specific time points before and after treatment. This ultrasound mode provides high-resolution and sensitive detection of slow, low-volume blood flow, enabling accurate assessment of microvascular perfusion within tumor tissues. MFI was performed using HD mode with grayscale gain set at 60%, MFI gain set at 51%, afterglow set to medium, and mechanical index adjusted to 0.7. The EMI of the tumor region was subsequently analyzed using QLAB software. To further quantify perfusion dynamics, CEUS was performed before and after UTMC treatment using the same ultrasound platform. CEUS imaging was performed in C-Pen mode with the following settings: contrast Side/Side, ultrasound gain at 63%, dynamic range at 50, and mechanical index adjusted to 0.06. For CEUS, following i.v. injection of microbubbles, time–intensity curves were generated, and peak intensity and AUC were calculated using QLAB software to assess tumor blood perfusion before and after UTMC treatment.

Intratumoral *p*O_2_ was measured using an OxyLite Pro tissue oxygen pressure monitor (Oxford Optronix) according to the manufacturer’s instructions. Briefly, a sterile fiber-optic oxygen probe was inserted into the central region of the tumor under anesthesia, and real-time *p*O_2_ values were continuously recorded for up to 20 minutes before and after treatment.

Twelve hours after treatment, mice were euthanized under anesthesia, and tumor tissues were collected. Immunohistochemical staining was performed to assess HIF-1A (anti–HIF-1A, 1:200; BF8002, Affinity) expression in the tumor region. In addition, tumor lysates were prepared for Western blot analysis to quantify HIF-1A protein expression. Western blotting was conducted as described in *Western blotting* above.

### Evaluation of antitumor effects of UTMC combined with anti–PD-L1 therapy in murine TNBC models.

Seven days after tumor establishment, mice were randomly divided into 6 groups (*n* = 5 per group) and received the following treatments: saline (control), ultrasound (25 mW/cm^2^), microbubbles (100×), UTMC, anti–PD-L1 (5 mg/kg; 124339, BioLegend), and anti–PD-1 plus UTMC (anti–PD-L1 was injected immediately after UTMC). LIPUS was applied locally to the tumor 5 seconds after saline injection (ultrasound group) or microbubble injection (UTMC group) and was performed continuously for 10 minutes in both conditions. The acoustic parameters of the therapeutic transducer were 1 MHz, 10 % duty cycle, 100 Hz pulse repetition frequency, and intensity of 25 mW/cm^2^. Anti–PD-L1 was administered via i.p. injection, microbubbles via i.v., and ultrasound irradiation was localized to tumor sites. Treatments were conducted every 2 days. Mouse body weights and tumor volumes were recorded every 3 days, beginning 1 day before the initial treatment. On day 18, mice in each group were anesthetized, sacrificed, and tumor tissues were harvested.

Immunofluorescent staining for α-SMA (1:2000; ET1607-53, HUABIO), CD31 (1:2000; ab281583, Abcam), NG2 (1:2000; A24955, ABclonal), VE-cadherin (1:500; ab318152, Abcam), and CD31 (1:2000; GB113151, ServiceBio) in tumor tissues was performed to assess tumor vasculature changes after treatment. Immunofluorescent staining for HIF-1A (1:200; BF8002, Affinity) and PD-L1 (1:200; HA722184, HUABIO) was used to evaluate hypoxia status and PD-L1 expression within tumors. Immunofluorescent staining for Icam-1 (1:5000; GB11106, ServiceBio), Vcam-1 (1:2000; GB153376, ServiceBio), Cxcl9 (1:2000; ab320827, Abcam), and CD31 (1:2000; GB113151, ServiceBio) was performed to investigate vasculature-associated immune trafficking signals. Immunofluorescence experiments also utilized DAPI (C1006, Beyotime) to stain nuclei.

To examine the effects of each treatment on immune cell infiltration in the TME, 120 mg of tumor tissue was processed into single-cell suspensions through enzymatic digestion. Cells were subsequently blocked with anti-CD16/anti-CD32 antibody to reduce nonspecific Fc receptor–mediated binding and stained following the manufacturer’s protocol. Populations including cytotoxic T lymphocytes (CD3^+^CD8^+^Tim-3^+/–^), helper T lymphocytes (CD3^+^CD4^+^Foxp3^–^), Tregs (CD3^+^CD4^+^Foxp3^+^), M1 macrophages (CD11b^+^F4/80^+^CD86^hi^CD206^lo^), and M2 macrophages (CD11b^+^F4/80^+^CD86^lo^CD206^hi^) were detected using an ACEA NovoCyte 3110 flow cytometer and analyzed using FlowJo software (version 10.8.0). The gating strategy for flow cytometry analysis is illustrated in Supplemental Figure 5. All antibodies and the viability dye were used at the highest concentrations recommended by the manufacturers. The following antibodies and reagents were used: Zombie Green (423111, BioLegend), anti-CD16/CD32 (101319, BioLegend), CD3-APC/Cy7 (100221, BioLegend), CD4-PerCP/Cy5.5 (116011, BioLegend), CD8-BV570 (100739, BioLegend), Tim-3-BV650 (416-5870-82, eBioscience), CD11b-BV785 (101243, BioLegend), Foxp3-BV421 (126419, BioLegend), CD86-PE (12-0862-82, eBioscience), and CD206-PE/Cy7 (141719, BioLegend).

Multiplex immunofluorescent staining for CD206 (1:1500; 24595, Cell Signaling Technology), F4/80 (1:1000; 70076, Cell Signaling Technology), CD8 (1:3000; 217344, Abcam), and Foxp3 (1:600; 12653, Cell Signaling Technology) along with DAPI staining was also employed to evaluate T cells and M2 macrophage infiltration within tumor regions. Additionally, tumor concentrations of TNF-α (SEKM-0034), IFN-γ (SEKM-0031), and IL-6 (SEKM-0007) were measured using ELISA kits (Solarbio).

### Statistics.

For bioinformatics analyses, all statistical tests and part of the data visualizations were performed using R software (version 4.5.0). Differences between groups for continuous variables were evaluated using the Wilcoxon rank-sum test (Mann-Whitney *U* test). For biological experiments, quantitative data are expressed as mean ± SD. An unpaired 2-tailed Student’s *t* test was applied for comparisons between 2 groups. Comparisons among multiple groups were performed using 1-way or 2-way analysis of variance (ANOVA) followed by Tukey’s or Šidák’s post hoc test. Statistical analyses for biological data were conducted using GraphPad Prism software (version 10.1.0). In all analyses, a *P* value of less than 0.05 was considered statistically significant. All experiments were performed with at least 3 independent replicates unless otherwise indicated.

### Study approval.

All animal experiments were performed under the Guidelines for Care and Use of Laboratory Animals of Drug Safety Evaluation Center of Harbin Medical University. All animal experiments were approved by the Medical Ethics Committee of First Affiliated Hospital of Harbin Medical University (no. 2023133).

### Data availability.

All data sets generated or analyzed during this study have been included in this manuscript, and values for all data points in graphs are reported in the Supporting Data Values file.

## Author contributions

ZZ and LB contributed equally to this work. ZZ, YJ, and JW designed research studies. ZZ and LB wrote the manuscript. CW and YJ supervised the project. ZZ, LB, SL, JW, and SW conducted the biological experiments and analyzed data. YL, ZZ, and SW performed the bioinformatics and statistical analyses. All authors read and approved the final manuscript.

## Conflict of interest

The authors have declared that no conflict of interest exists.

## Funding support

- National Natural Science Foundation of China grants 82503713 (to ZZ) and 81671697 (to CW).
- Key Project of Natural Science Foundation of Heilongjiang Province grant ZD2023H002 (to YJ).
- Heilongjiang Province “Double First Class” Discipline Collaborative Innovation Achievement Cultivation Project grant LJGXCG2024-P12 (to YJ).
- First Affiliated Hospital of Harbin Medical University Fund for Excellent Young Scholars grant 2024YQ02 (to JW).

## Supplementary Material

Supplemental data

Unedited blot and gel images

Supporting data values

## Version 1 04/07/2026

In-Press Preview

## Version 2 05/22/2026

Electronic publication
