# Supplementary material for: Ultrasound-targeted microbubble cavitation enhances anti–PD-L1 therapy in TNBC via eNOS-mediated reoxygenation
Source: JCI Insight. 2026 Apr 7;11(10):e198349. doi: 10.1172/jci.insight.198349 (PMC13232718; doi:10.1172/jci.insight.198349)
Supplement: Supplemental data [file jciinsight-11-198349-s039.pdf]

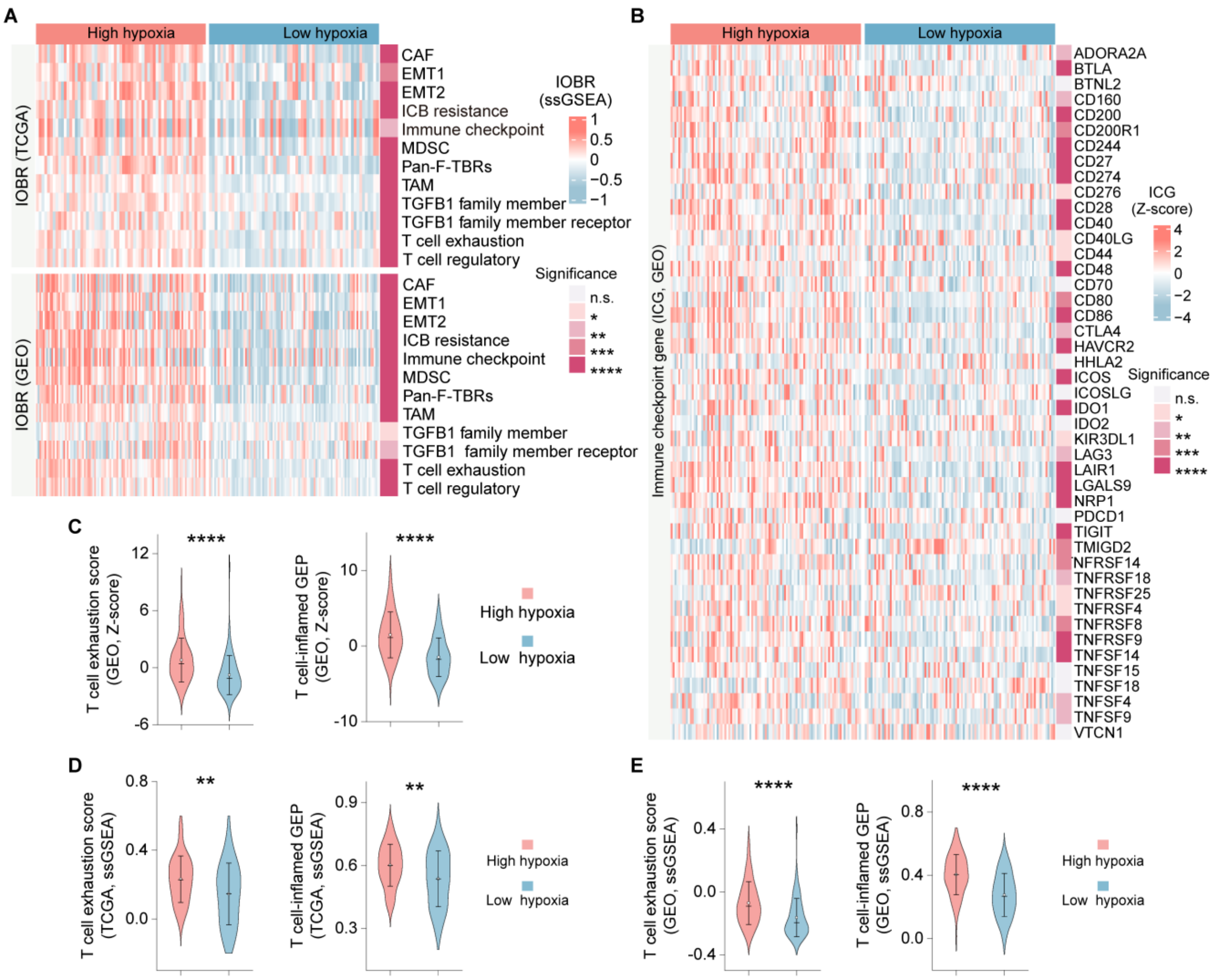

Supplemental Figure 1. Immunological characterization of patients with TNBC stratified by hypoxia status. (**A**) Heatmap depicting IOBR-derived functional signatures in TNBC samples stratified by high- and low-hypoxia status from the TCGA and GEO cohorts. (**B**) Heatmap depicting normalized expression of 45 immune checkpoint genes (ICGs) in TNBC samples stratified by high- and low-hypoxia status from the GEO dataset. (**C-E**) Comparison of T cell exhaustion scores and T cell inflamed gene expression profile (GEP) between high- and low-hypoxia TNBC groups in the TCGA and GEO dataset. Statistical analyses were performed using Wilcoxon's rank-sum test. n.s.: no significance; *P < 0.05; **P < 0.01; ***P < 0.001; ****P < 0.0001.

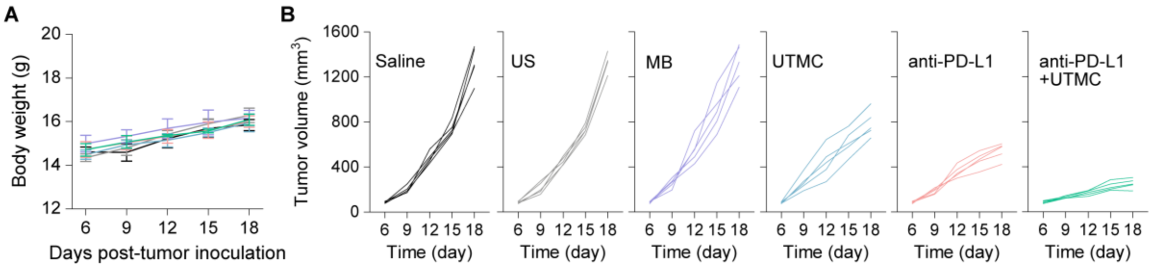

Supplemental Figure 2. Body weight and tumor growth dynamics in 4T1 tumor-bearing mice following the indicated treatments. (**A**) Body weight changes of 4T1 tumor-bearing mice following various treatments. (**B**) Individual tumor growth curves in mice subjected to different treatments (n = 5 per group).

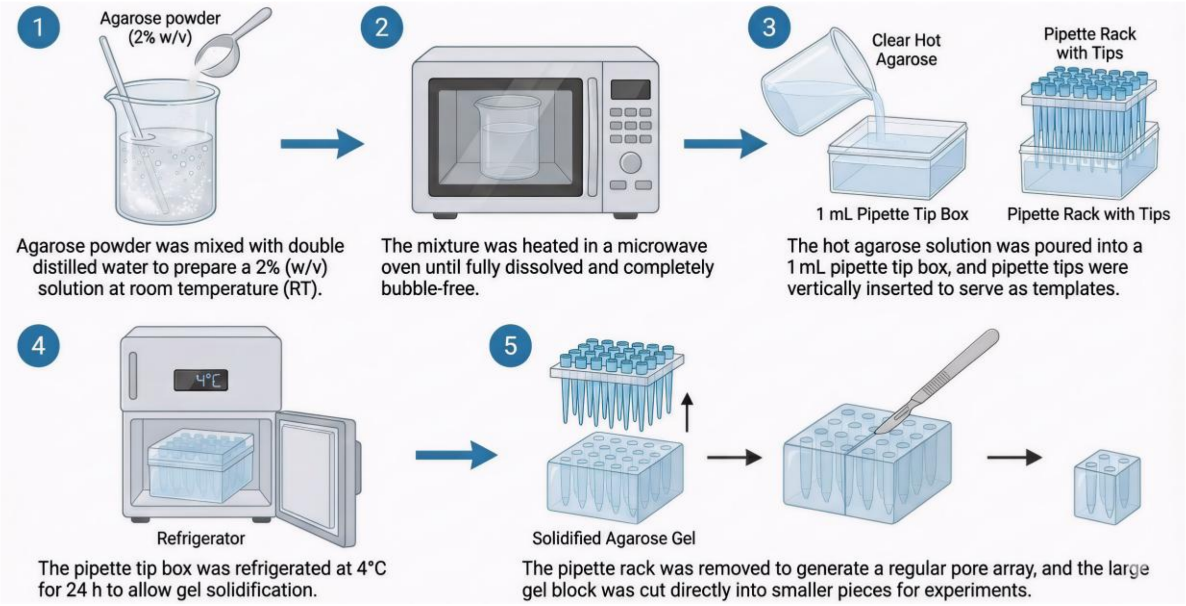

Supplemental Figure 3. Agarose phantom preparation workflow.

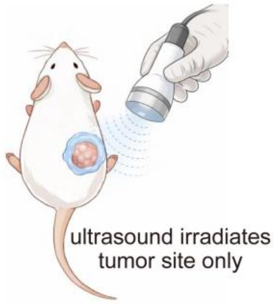

Supplemental Figure 4. Schematic illustration of ultrasound irradiation treatment.

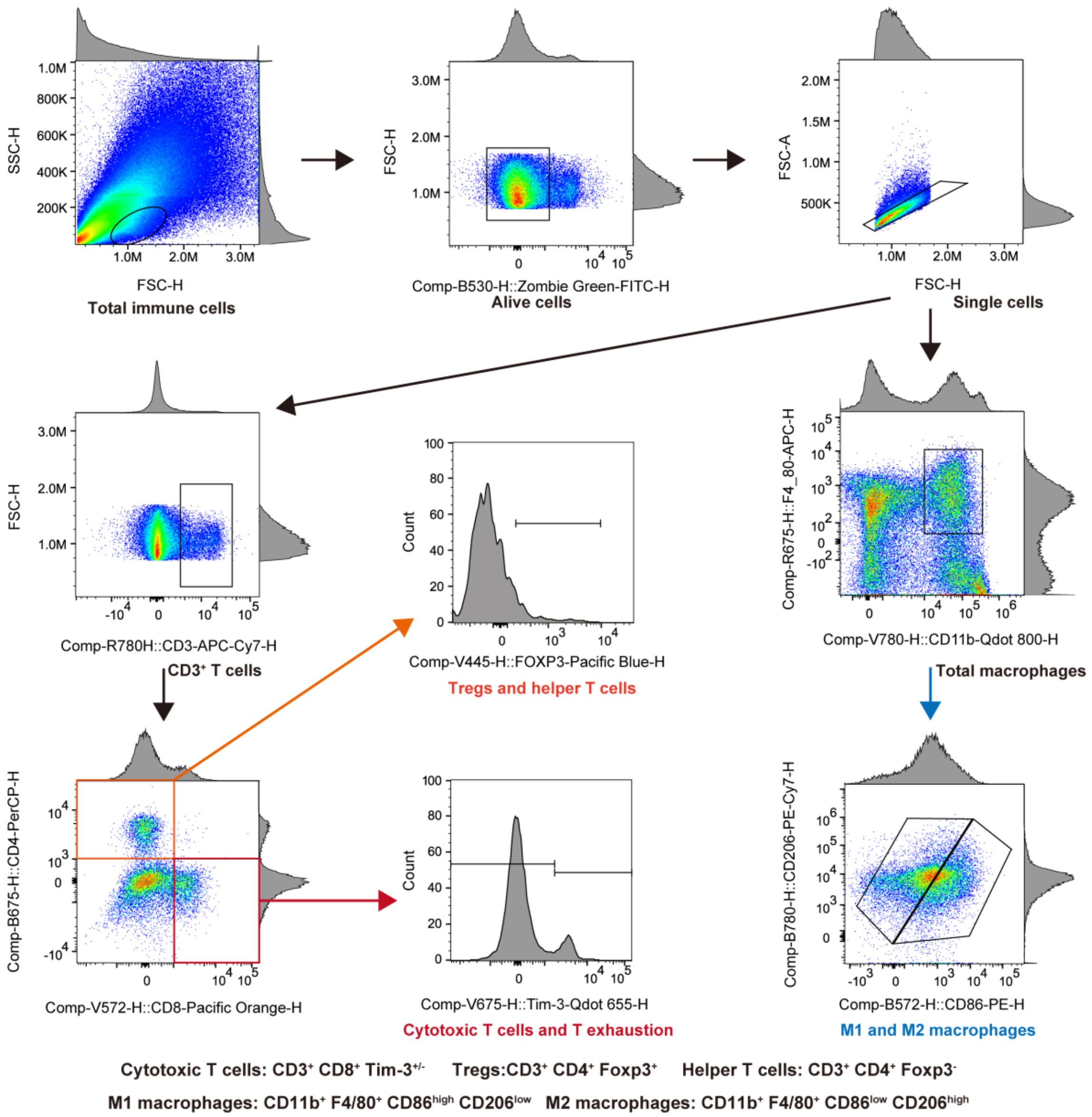

Supplemental Figure 5. Gating strategy for flow cytometry analysis of tumor-infiltrating immune cells in vivo.

Variables	Type	HR	*P* value	SE	Low	High
Hypoxia	OS	0.969	0.947	0.466	0.39	2.4
Hypoxia	DSS	1.06	0.916	0.56	0.35	3.2
Hypoxia	PFI	0.619	0.264	0.43	0.27	1.4

Supplemental Table 1. Cox proportional hazards analysis evaluating the association between hypoxia status and clinical outcomes in patients with TNBC from TCGA. OS, overall survival; DSS, disease-specific survival; PFI, progression-free interval; HR, hazard ratio; SE, standard error.

Gene	Trimer sequence (5'-3')	Primer direction
GNAQ	TGGGTCAGGATACTCTGATGAAG	forward primer
	TGTGCATGAGCCTTATTGTGC	reverse primer
GNA11	GGCTTCACCAAGCTCGTCTAC	forward primer
	CACTGACGTACTGATGCTCG	reverse primer
GNAS	TGCCTCGGGAACAGTAAGAC	forward primer
	GCCGCCCTCTCCATTAAAC	reverse primer

Supplemental Table 2. Primer sequences used for quantitative real-time PCR (qRT-PCR).

Gene	Target Sequence (5'-3')	siRNA strand
GNAQ	CCACAGACACCGAGAAUAUTT	Sense
	AUAUUCUCGGUGUCUGUGGTT	antisense
GNAQ	CAAUAAGGCUCAUGCACAATT	Sense
	UUGUGCAUGAGCCUUAUUGTT	antisense
GNAQ	CCACAGGGAUCAUCGAAUATT	Sense
	UAUUCGAUGAUCCCUGUGGTT	antisense
GNA11	GGGAGGUGGACGUGGAGAATT	Sense
	UUCUCCACGUCCACCUCCCTT	antisense
GNA11	GGGAAGAGCACGUUCAUCATT	Sense
	UGAUGAACGUGCUCUUCCCTT	antisense
GNA11	AGGACCUGCUGGAGGACAATT	Sense
	UUGUCCUCCAGCAGGUCCUTT	antisense
GNAS	GCAUGUUAAUGGGUUUAAUTT	Sense
	AUUAAACCCAUUAACAUGCTT	antisense
GNAS	CUGAAAGAGGCGAUUGAAATT	Sense
	UUUCAAUCGCCUCUUUCAGTT	antisense
GNAS	GAAGAUUGAGGACUACUUUTT	Sense
	AAAGUAGUCCUCAAUCUUCTT	antisense

Supplemental Table 3. Sequences of siRNAs used for gene knockdown experiments.
