# Supplementary material for: Ultrasound-targeted microbubble cavitation enhances anti–PD-L1 therapy in TNBC via eNOS-mediated reoxygenation
Source: JCI Insight. 2026 Apr 7;11(10):e198349. doi: 10.1172/jci.insight.198349 (PMC13232718; doi:10.1172/jci.insight.198349)

Full unedited gel for Figure 3D

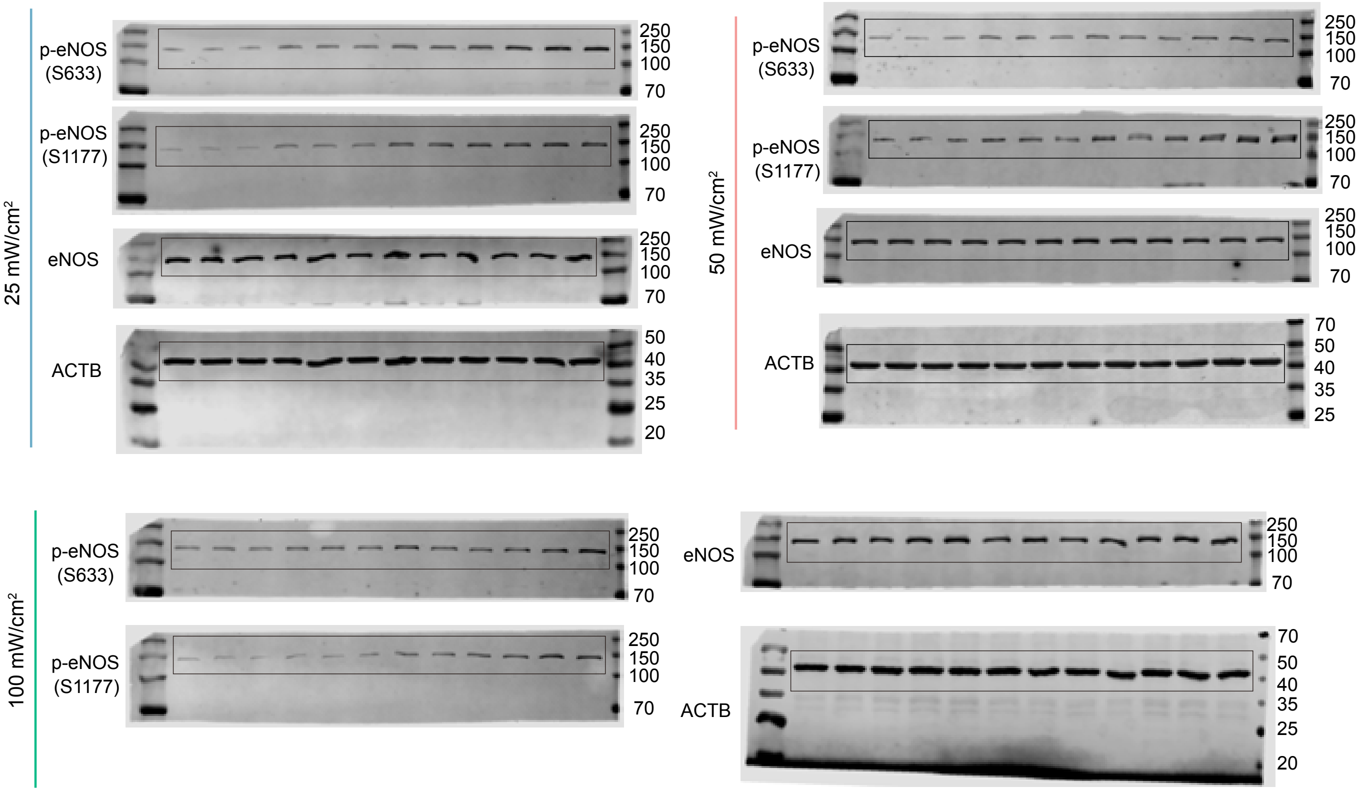

Full unedited gel for Figure 4A

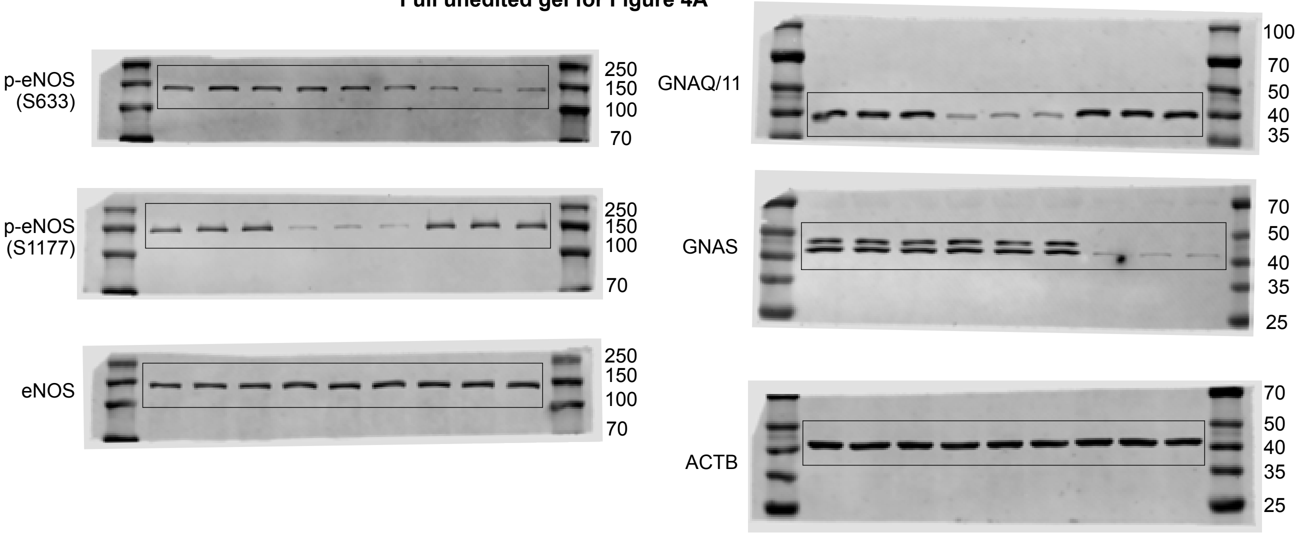

Full unedited gel for Figure 4K

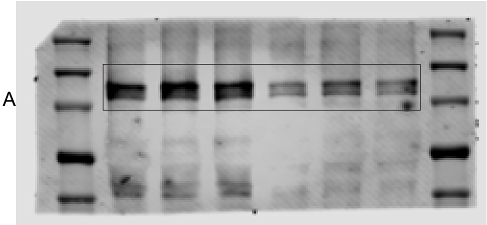

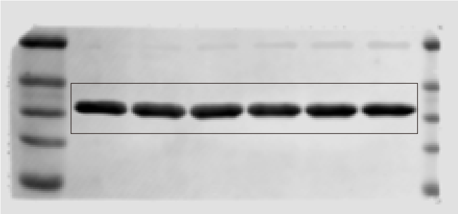

Supplement: Unedited blot and gel images [file jciinsight-11-198349-s040.pdf]
